# Inhibition of SARS‐CoV‐2‐mediated thromboinflammation by CLEC2.Fc

**DOI:** 10.15252/emmm.202216351

**Published:** 2023-05-22

**Authors:** Pei‐Shan Sung, Cheng‐Pu Sun, Mi‐Hua Tao, Shie‐Liang Hsieh

**Affiliations:** ^1^ Genomics Research Center Academia Sinica Taipei Taiwan; ^2^ Institute of Biomedical Sciences Academia Sinica Taipei Taiwan; ^3^ Immunology Research Center National Health Research Institutes Zhunan Taiwan; ^4^ Institute of Clinical Medicine National Yang Ming Chiao Tung University Taipei Taiwan; ^5^ Department of Medical Research Taipei Veterans General Hospital Taipei Taiwan

**Keywords:** CLEC2, NET, platelet, SARS‐CoV‐2, thromboinflammation, Microbiology, Virology & Host Pathogen Interaction

## Abstract

Thromboinflammation is the major cause of morbidity and mortality in COVID‐19 patients, and post‐mortem examination demonstrates the presence of platelet‐rich thrombi and microangiopathy in visceral organs. Moreover, persistent microclots were detected in both acute COVID‐19 and long COVID plasma samples. However, the molecular mechanism of SARS‐CoV‐2‐induced thromboinflammation is still unclear. We found that the spleen tyrosine kinase (Syk)‐coupled C‐type lectin member 2 (CLEC2), which was highly expressed in platelets and alveolar macrophages, interacted with the receptor‐binding domain (RBD) of SARS‐CoV‐2 spike protein (SARS‐CoV‐2 RBD) directly. Unlike the thread‐like NETs, SARS‐CoV‐2‐induced aggregated NET formation in the presence of wild‐type (WT), but not CLEC2‐deficient platelets. Furthermore, SARS‐CoV‐2 spike pseudotyped lentivirus was able to induce NET formation via CLEC2, indicating SARS‐CoV‐2 RBD engaged CLEC2 to activate platelets to enhance NET formation. Administration of CLEC2.Fc inhibited SARS‐CoV‐2‐induced NET formation and thromboinflammation in AAV‐ACE2‐infected mice. Thus, CLEC2 is a novel pattern recognition receptor for SARS‐CoV‐2, and CLEC2.Fc and may become a promising therapeutic agent to inhibit SARS‐CoV‐2‐induced thromboinflammation and reduced the risk of post‐acute sequelae of COVID‐19 (PASC) in the future.

## Graphical abstract

CLEC2 interacts with SARS‐CoV‐2 spike RBD and is critical for SARS‐CoV‐2‐induced lung injury. In the presence of platelets, SARS‐CoV‐2 induces robust NET formation and cytokine release, while administration of CLEC2.Fc prevents platelet‐mediated enhancement of NET formation and cytokine storm via blocking interactions between CLEC2 and SARS‐CoV‐2 spike protein.

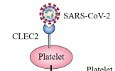

**The paper explained**

**Problem**

The mechanisms that contribute to SARS‐CoV‐2‐induced platelet activation and immunothrombosis are poorly understood. Despite the evidence for hyperactivation of platelets, microcoagulopathy, NETosis, and microclot formation in blood in SARS‐CoV‐2 infection, there has been limited advance in understanding the roles of platelets in SARS‐CoV‐2‐induced thromboinflammation, as well as the pattern recognition receptor for SARS‐CoV‐2‐induced platelet activation.

**Results**

We show that the Syk‐coupled C‐type lectin member 2 (CLEC2) serves as pattern recognition receptor for the receptor binding domain (RBD) of spike protein from various SARS‐CoV‐2 strains. While SARS‐CoV‐2 alone is a weak activator of NET formation, SARS‐CoV‐2‐activated platelets are potent enhancers of aggregated NET formation. While classical thread‐like NETs are adhesive, the aggregated NETs detach from glass plate and float in culture supernatant, suggesting that SARS‐CoV‐2 spike RBD engages CLEC2 to activate platelets, leading to immunothrombosis and microclots through floating aggregated NETs. Furthermore, *in vivo* experiments demonstrate that recombinant CLEC2.Fc fusion protein effectively inhibits SARS‐CoV‐2‐induced immunothrombosis, indicating that blocking the CLEC2‐mediated signaling pathway is promising for treating immunothrombosis caused by SARS‐CoV‐2.

**Impact**

Our discovery confirms the crucial involvement of CLEC2 in virus‐induced platelet hyperactivation and immunothrombosis, as evidenced by excessive NET formation and protease‐resistant microclots detected in COVID‐19 patient plasma. Moreover, we reported that aggregated NET formation was induced by extracellular vesicles (EVs) from COVID‐19 patients. These findings collectively highlight the significant role of hyperactivated platelets and platelet‐derived EVs in the pathogenesis of microangiopathy in COVID‐19 patients, providing new directions for developing therapeutic agents to treat diseases resulting from microangiopathy in the future.

## Introduction

The severe acute respiratory syndrome coronavirus 2 (SARS‐CoV‐2) is the etiological agent of coronavirus‐induced disease 19 (COVID‐19; Zhu *et al*, 2020). Similar to the severe acute respiratory syndrome coronavirus (SARS‐CoV; Li *et al*, 2003; Wang *et al*, 2004), SARS‐CoV‐2 binds angiotensin‐converting enzyme 2 (ACE2) via the receptor‐binding domain of spike protein (spike RBD) for cellular entry (Hoffmann *et al*, 2020; Yan *et al*, 2020). In addition, SARS‐CoV‐2 induces cytokine storm to cause acute respiratory distress syndrome (ARDS; Huang *et al*, 2020), and COVID‐19 patients often suffer from severe pulmonary inflammation with thrombotic complications, such as microangiopathy, pulmonary embolism (Klok *et al*, 2020), venous thrombosis (Wichmann *et al*, 2020), and cerebral infarction (Moshayedi *et al*, 2020; Oxley *et al*, 2020). Moreover, hyperactivated platelets and persistent microclots (comprising aggregated proteins resistant to trypsin digestion) were detected in both acute COVID‐19 and long COVID plasma samples (Pretorius *et al*, 2021; Wang *et al*, 2022). Thus, it is crucial to understand how SARS‐CoV‐2 induces microemboli in order to reduce the risk of PASC in SARS‐CoV‐2‐infected patients.

Recent studies demonstrate that platelets are hyperactivated (Comer *et al*, 2021; Leopold *et al*, 2021) and play critical roles in NET formation (Andrews *et al*, 2014; Gomez *et al*, 2020) and immunothrombosis in COVID‐19 patients (Grover & Mackman, 2018; Koupenova & Freedman, 2020). Moreover, extracellular vesicles (EVs) isolated from COVID‐19 patients expressed abundant markers characteristic of activated platelets. Incubation of COVID‐19 EVs induces aggregated NET formation and thromboinflammation via CLEC5A and TLR2, suggesting activated platelets contribute to the pathogenesis of COVID‐19 (Sung *et al*, 2022). However, the molecular mechanism of SARS‐CoV‐2‐induced platelet activation and thromboinflammation is still unclear. More and more evidence demonstrates that neutrophil extracellular traps (NETs) are not only a disease severity marker (Zuo *et al*, 2020), but also contribute to the pathogenesis of SARS‐CoV‐2‐induced multiple organ damage, including acute lung injury (Middleton *et al*, 2020; Yaqinuddin *et al*, 2020), neuroinflammation (Tomar *et al*, 2020), and vascular occlusion (Escalard *et al*, 2020; Leppkes *et al*, 2020; Makatsariya *et al*, 2020) in COVID‐19 patients. Compared to bacteria‐induced NET formation, the kinetics of virus‐induced NET formation is much slower. For examples, *Listeria monocytogenes* induces robust NET formation within 30 min (Chen *et al*, 2017), while dengue virus (DV) and human immunodeficiency virus (HIV) induce NET formation at 3 h and 24 h, respectively, post‐incubation with neutrophils (Saitoh *et al*, 2012; Sung *et al*, 2019). However, it is still unclear how SARS‐CoV‐2 induces NET formation.

CLEC2 is a spleen tyrosine kinase (Syk)‐coupled C‐type lectin and is highly expressed in platelets and alveolar macrophages (Colonna *et al*, 2000; Suzuki‐Inoue *et al*, 2006). Interactions of CLEC2 with its endogenous ligand podoplanin is crucial in the development of vascular vessels and cerebrovascular integrity (Osada *et al*, 2012; Lowe *et al*, 2015). Moreover, administration of CLEC2.Fc fusion protein (comprising the extracellular domain of CLEC2 and Fc portion of human IgG1) attenuates TNF production and inflammatory macrophage accumulation in an LPS‐induced peritonitis model, suggesting blockade of CLEC2 and its endogenous ligand podoplanin is able to attenuate inflammation *in vivo* (Bourne *et al*, 2021). CLEC2 was shown to associate with DC‐SIGN to capture HIV, thereby facilitated HIV dissemination in infected patients (Chaipan *et al*, 2006). Recently, it has been demonstrated that platelets were activated by DV via CLEC2, thereby released EVs to enhance NET formation and proinflammatory cytokine release (Sung *et al*, 2019). However, DV does not interact with CLEC2 directly; thus, DV may be captured by DC‐SIGN to engage CLEC2, thereby activates platelets to enhance NET formation (Sung *et al*, 2019). As NETs play critical roles in thromboemoboli formation (Escalard *et al*, 2020; Leppkes *et al*, 2020; Makatsariya *et al*, 2020), we are interested to understand whether CLEC2 contributes to SARS‐CoV‐2 induced NET formation.

Here we report that CLEC2 interacted with the RBDs of several SARS‐CoV‐2 strains, but not SARS‐CoV RBD. SARS‐CoV‐2 RBD engaged CLEC2 directly to activate platelets, thereby induced robust aggregated NET formation. Blockade of CLEC2 attenuated SARS‐CoV‐2‐induced NET formation and proinflammatory cytokine release *in vitro*, and administration of CLEC2.Fc not only abolished NET formation, but also attenuated cell infiltration and inflammation in SARS‐CoV‐2‐infected AAV‐ACE2 mice. Thus, CLEC2 is a novel pattern recognition receptor for SARS‐CoV‐2, and CLEC2.Fc may become a promising therapeutic agent to protect COVID‐19 patients from thromboinflammation and reduce PASC in the future.

## Results

### Interaction between CLEC2 and SARS‐CoV‐2 RBD

Our previous work demonstrated that CLEC2 was critical for DV‐induced platelet activation, and blockade of CLEC2 abolished platelet‐mediated enhancement of NET formation (Sung *et al*, 2019). However, DV did not bind CLEC2 directly, and DV‐mediated CLEC2 activation was dependent on its binding to a multivalent hetero‐complex comprising DC‐SIGN and mannose receptor (Chen *et al*, 2008; Lo *et al*, 2016). In addition, several C‐type lectins have been demonstrated to play critical roles in SARS‐CoV‐2‐induced lethality (Chen *et al*, 2008, 2012; Sung & Hsieh, 2019); thus, we asked whether CLEC2 was involved in SARS‐CoV‐2‐induced thromboinflammation.

**Figure 1:**
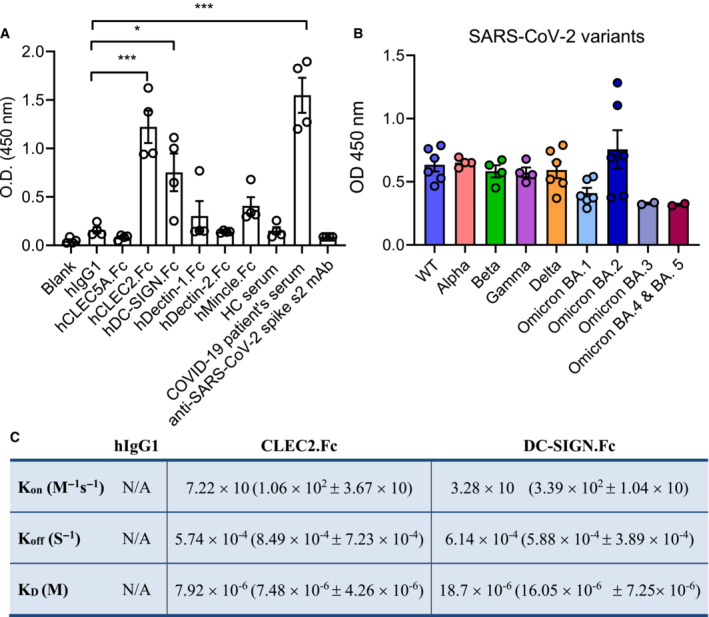
Human CLEC2 specifically binds to the RBD of SARS‐CoV‐2 variants A, B
Interaction between several human C‐type lectins (50 μg/ml) and recombinant SARS‐CoV‐2 RBD (50 μl/well) was determined by ELISA (A), and the interactions of hCLEC2.Fc with SARS‐CoV‐2 RBDs from various strains (B).C
The binding affinity of SARS‐CoV‐2‐spike RBD and human CLEC2.Fc fusion protein was measured by biolayer interferometry. K_a_: Rate constants for association. K_d_: Rate constants for dissociation. K_D_: Equilibrium dissociation constant. Data information: Data in (A) is presented as mean ± SEM, each experiment was repeated independently at least four times. *P < 0.05, ***P < 0.001 (Student's *t*‐test). Source data are available online for this figure.

As DC‐SIGN was shown to interact with SARS‐CoV‐2 RBD specifically (Amraei *et al*, 2021), we asked whether CLEC2 also interacted with SARS‐CoV‐2 RBD. To address this question, we generated human CLEC2.Fc fusion protein (Chen *et al*, 2008; Sung *et al*, 2019) to test its binding ability to recombinant SARS‐CoV RBD and SARS‐CoV‐2 RBD, respectively. We found that CLEC2 bound SARS‐CoV‐2 RBD (Fig 1A) specifically, while its binding to SARS‐CoV RBD is relatively weak (blue column, Appendix Fig S1A). To confirm this observation, we purchased monomeric SARS‐CoV‐2 RBD of several SARS‐CoV‐2 strains (WT, alpha, beta, gamma, delta, and omicron variants from Acro Biosystems) to test their interactions with CLEC2. We found that CLEC2.Fc not only bound wild‐type (WT) SARS‐CoV‐2 RBD, but also bound the RBDs of all other SARS‐CoV‐2 strains (Fig 1A and B, Appendix Fig S1B). In addition to monomeric RBD, CLEC2.Fc also bound WT SARS‐CoV‐2 trimeric RBD (Appendix Fig S1C). We further measured the binding affinity between CLEC2.Fc/SARS‐CoV‐2 RBD and DC‐SIGN.Fc/SARS‐CoV‐2 RBD, respectively, by bio‐layer interferometry (BLI). We found that the affinity between SARS‐CoV‐2 RBD and CLEC2 was 7.92 × 10^−6^ (M; Fig 1C), which was similar to that between SARS‐CoV‐2 RBD and DC‐SIGN (18.7 × 10^−6^ (M)) and slightly higher than that between CLEC2 and its endogenous ligand podoplanin (24.5 × 10^−6^ (M); Christou *et al*, 2008). These observations suggest that CLEC2 is a novel pattern recognition receptor for SARS‐CoV‐2.

**Figure 2:**
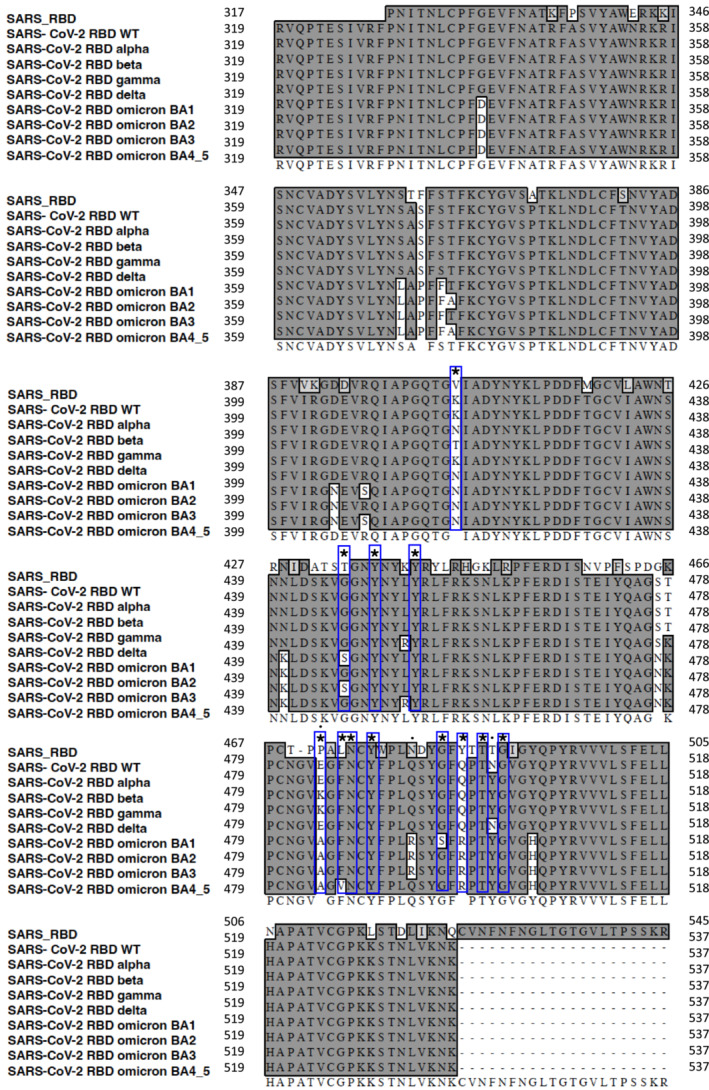
Sequence alignment of SARS‐CoV‐2 RBD strains and SARS‐CoV RBD The RBD sequences of SARS‐CoV‐2 (Taiwan strain, WT, alpha, beta, gamma, delta, omicron BA.1, omicron BA.2, omicron BA.3, omicron BA.4 & 5) and of SARS‐CoV were aligned with Clustal program under MacVector software (version 17.5.6). The star sign denotes the amino acid residues (K417, G446, Y449, Y453 E484, F486, N487, Y489, G496, Q498, T500, and G502) critical for SARS‐CoV‐2 binding to ACE2 (Hoffmann *et al*, 2020). The dot sign in black denotes the distinct amino acid residues in RBDs among SARS‐CoV‐2 strains. WT: wild type SARS‐CoV‐2 RBD.

Multiple‐sequence alignment reveals the most divergent region between SARS‐CoV RBD (a.a. 426–490) and SARS‐CoV‐2 RBD (a.a. 438–506; Fig 2) overlaps with the receptor‐binding motif (RBM) of SARS‐CoV‐2 RBD (a.a. 437–508). This region (a.a. 438–503) includes 12 amino acid residues (^**★**^K_417_, ^**★**^G_446_, ^**★**^Y_449_, ^**★**^Y_453_, ^**★**^E_484_, ^**★**^F_486_, ^**★**^N_487_, ^**★**^Y_489_, ^**★**^G_496_, ^**★**^Q_498_, ^**★**^T_500_, ^**★**^G_502,_ in blue boxes) critical for SARS‐CoV‐2 binding to ACE2 (Walls *et al*, 2020), as well as nine amino acid residues (^•^N/K_440_, ^•**★**^G/S_446_, ^•^K/R_452_, ^•^S/N_477_, ^•^T/K_478_, ^•^* E /K/A_484_, ^•^Q/R_493_, ^•^Y/N_501_, ^•^Y/H_505_) which distinguish different SARS‐CoV‐2 strains. Because CLEC2 interacts with all the nine SARS‐CoV‐2 RBDs (Fig 1B), this observation suggests that these nine amino acid residues and the 12 highly conserved amino acid residues in RBD domain may not affect CLEC2 binding to SARS‐CoV‐2 RBD. However, other amino acids (_441_ATS_443_, _458_NVPFSPDGK_446_, _469_TPPAL_473_, _498_ND_499_, _503_YT_504_) located in SARS‐CoV RBD may contribute to the differential binding between SARS‐CoV BRD and SARS‐CoV‐2 RBD to CLEC2.

### SARS‐CoV‐2 activates platelet via CLEC2 to enhance NET formation

**Figure 3:**
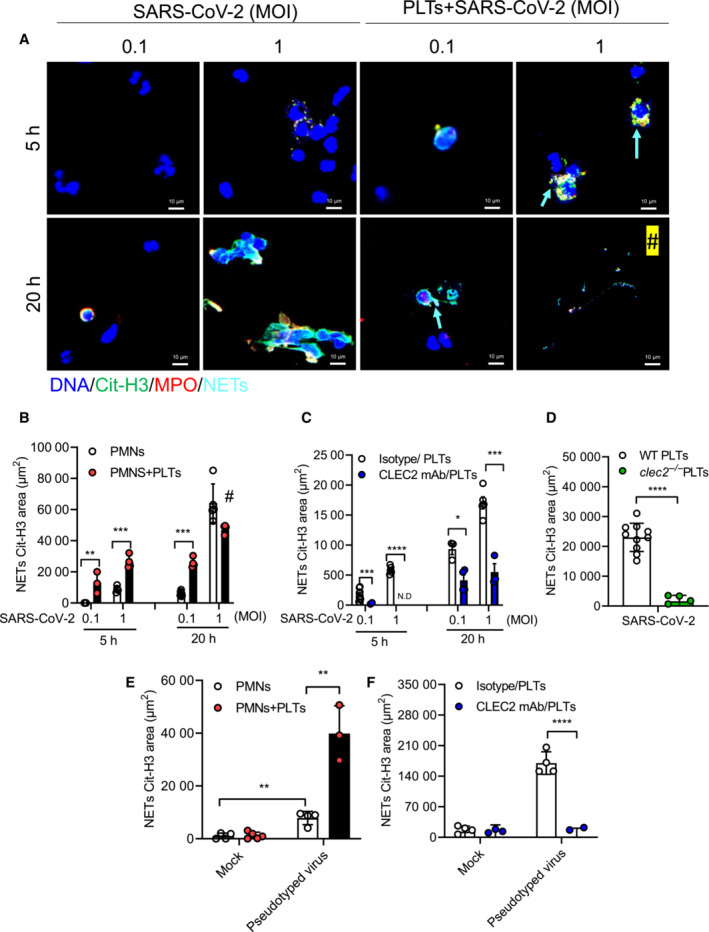
SARS‐CoV‐2 activates platelet CLEC2 to induce NET formation via spike RBD Human neutrophils (PMNs, 4 × 10^5^/ml) were incubated with SARS‐CoV‐2 (MOI = 0.1 and 1) with or without autologous platelets (PLTs, 4 × 10^6^/ml) for 5 and 20 h at 37°C.A
The image of NETs (arrowheads: aggregated NETs).B
Quantitation of NET area by citrullinated Histone H3 (Cit‐H3) area.C
Human platelets were pretreated with anti‐CLEC2 mAb (AYP1, 2.5 μg/ml) or isotype control for 15 min at room temperature before incubation with neutrophils and SARS‐CoV‐2 stimulation.D
Neutrophils (4 × 10^5^/ml) from wild type (WT) mice were incubated with SARS‐CoV‐2 (MOI = 1) in the presence of WT platelets (4 × 10^6^/ml) or *clec2* ^*−/−*^ platelets (4 × 10^6^/ml) for 5 h at 37°C.E, F
Human neutrophils were stimulated with SARS‐CoV‐2 spike RBD pseudotyped virus (pseudotyped virus; MOI = 0.1) in presence of autologous platelets at 37°C for 3 h (E). Platelets were incubated with anti‐CLEC2 mAb (2.5 μg/ml) at room temperature for 15 min before incubation with neutrophils and pseudotyped virus stimulation at 37°C for 3 h (F). The level of NET formation was determined using Cit‐H3 area (μm^2^). Data information: Data are mean ± SD from at least three independent experiments. *P < 0.05, **P < 0.01, ***P < 0.001, ****P < 0.0001 (Student's *t*‐test). #: The less NETs attached to the plate at 20 h (in the presence of platelets) are due to the robust aggregated NETs which were detached from the culture plate. Source data are available online for this figure.

We further asked whether platelets were able to enhance SARS‐CoV‐2‐induced NET formation. To address this question, human neutrophils were incubated with SARS‐CoV‐2 in the presence or absence of autologous platelets. At lower virus titer (MOI = 0.1), SARS‐CoV‐2 alone was barely able to induce NET formation at 5 h and 20 h post‐incubation with neutrophils, while obvious NET formation was observed at higher viral titer at 20 h post‐incubation (MOI = 1; Fig 3A and white column in Fig 3B). In the presence of platelets, even lower viral titer (MOI = 0.1) can induce obvious NET formation at 5 h and 20 h post‐incubation (right, Fig 3A and black column in Fig 3B), while higher viral titer (MOI = 1) induced aggregated NET formation at 5 h post‐incubation (MOI = 1). Interestingly, robust aggregated NETs detached from glass plate, leading to less NET formation attached on the glass platelet (#, Fig 3A and B) and floated in the culture medium (Appendix Fig S2 (II)) at 20 h post‐incubation. To further support this observation, we detected DNA‐MPO‐Elastase complex in culture supernatant by ELISA using PMA as positive control. Even though the NETs sticked to glass plates is less than PMA at 3 h post‐incubation (right lower panels, Appendix Fig S3A and B), the level of DNA‐MPO‐Elastase complex in culture supernatant is similar after incubation with PMA and pseudotyped virus/platelet mixture (Appendix Fig S3C). These observations suggest that platelets not only enhance SARS‐CoV‐2‐induced NET formation, but also induce robust aggregated NETs formation and abundant DNA‐MPO‐Elastase complex in culture supernatant.

We then asked whether blockade of CLEC2 had any inhibitory effect to platelet‐enhanced NET formation. While SARS‐CoV‐2‐induced NET formation was observed in a dose‐dependent manner, NET formation was inhibited efficiently by anti‐CLEC2 mAb (Fig 3C, Appendix Fig S2 (III)). Because SARS‐CoV‐2 spike‐RBD also bound mouse CLEC2 (Appendix Fig S4), we compared the effect of wild‐type (WT) and *clec2* ^−/−^ platelets in SARS‐CoV‐2‐induced NET formation. We found that NET formation was almost undetectable in the presence of *clec2* ^−/−^ platelets (green column, Fig 3D), and this observation indicated that CLEC2 was critical in platelet‐mediated enhancement of NET formation. To further confirm the role of SARS‐CoV‐2 spike protein in SARS‐CoV‐2‐induced NET formation, we generated a SARS‐CoV‐2 spike pseudotyped lentivirus to address this question. While little amount of NET was observed by virus alone, NET formation was greatly enhanced in the presence of platelets (Fig 3E). Moreover, anti‐CLEC2 mAb inhibited NET formation induced by SARS‐CoV‐2 spike pseudotyped lentivirus (Fig 3F). This observation suggests that SARS‐CoV‐2 spike RBD activates platelet via CLEC2 to enhance NET formation.

### CLEC2.Fc is able to inhibit SARS‐CoV‐2‐induced thromboinflammation

**Figure 4:**
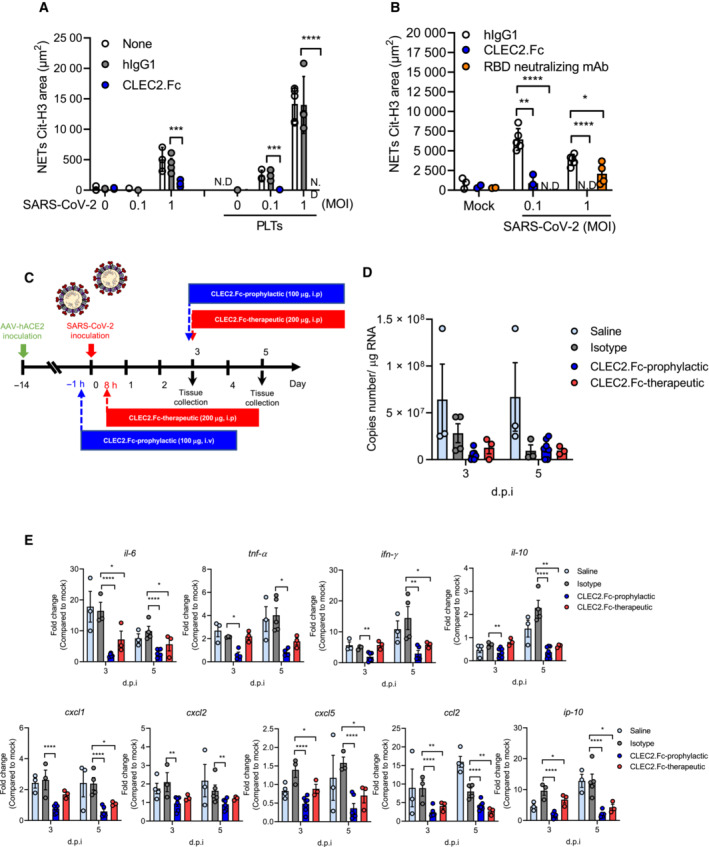
The prophylactic and therapeutic effects of CLEC2.Fc in SARS‐CoV‐2‐challenged AAV‐hACE2 mice A, B
Human neutrophils were stimulated with SARS‐CoV‐2 (MOI = 0.1 and 1) at 37°C in the presence of isotype control (hIgG1, 3 μg/ml) or human CLEC2.Fc (3 μg/ml) and with or without autologous platelets for 5 h (A) or with or without anti‐SARS‐CoV‐2 spike RBD antibody (3 μg/ml) for 20 h (B). Data are means ± SD from at least three independent experiments. *P < 0.05, **P < 0.01, ***P < 0.001, ****P < 0.0001 (Student's *t*‐test).C–E
C57BL/6 mice were inoculated with AAV‐hACE2 at day 14 before the SARS‐CoV‐2 challenge. For the prophylactic use of hCLEC2.Fc, mice were intravenously (i.v.) injected with CLEC2.Fc (100 μg/mouse) then intratracheally injected with SARS‐CoV‐2 (8 × 10^4^ PFU). For the therapeutic use of hCLEC2.Fc, mice were intratracheally inoculated with SARS‐CoV‐2 (8 × 10^4^ PFU) and intraperitoneally injected with CLEC2.Fc (100 μg/per mice). Lung tissues were collected at day 3 day and day 5 post‐infection. (C) The expression levels of viral copy number (D), cytokines and chemokines in lung were measured by qPCR (E). Data information: Data are mean ± SEM from two independent experiments (saline: *n* = 3 for 3 d.p.i., *n* = 4 for 5 d.p.i.; isotype: *n* = 3 for 3 d.p.i., *n* = 4 for 5 d.p.i.; prophylactic treatment of CLEC2.Fc treatment: *n* = 5 for 3 d.p.i. and 5 d.p.i.; therapeutic treatment of CLEC2.Fc: *n* = 3 for 3 d.p.i. and 5 d.p.i.). *P < 0.05, **P < 0.01, ***P < 0.001, ****P < 0.0001 (two‐way ANOVA). ‘d.p.i.’ stands for ‘day post‐infection’. Source data are available online for this figure.

As CLEC2.Fc interacted with SARS.CoV‐2 RBD directly, we asked whether CLEC2.Fc was able to inhibit SARS.CoV‐2‐induced NET formation *in vitro*. We found that CLEC2.Fc not only inhibited SARS‐CoV‐2‐induced NET formation (left, Fig 4A) but also suppressed platelet‐mediated enhancement of NET formation *in vitro* (right, Fig 4A). Moreover, CLEC2.Fc had similar effect as anti‐SARS‐CoV‐2 spike‐RBD mAb to inhibit NET formation (Fig 4B), but CLEC2.Fc was barely able to inhibit SARS‐CoV‐2 infection in Vero cells (Appendix Table S1). These observations suggest that the contact region of SARS‐CoV‐2 spike RBD for virus entry is different from that for platelet activation.

In order to understand whether CLEC2.Fc had protective effects in SARS‐CoV‐2‐induced thromboinflammation *in vivo*, we inoculated SARS‐CoV‐2 to the AAV‐ACE2‐infected mice to address this question, as this mouse model mimics the pathophysiology of COVID‐19 patients (Sun *et al*, 2021). In the prophylactic model (blue bars, Fig 4C), mice were injected with CLEC2.Fc (100 μg) at 60 min before intravenous inoculation of SARS‐CoV‐2 (lower blue bar, Fig 4C), followed by a second dose of CLEC2.Fc (100 μg) at day 3 post‐virus infection (upper blue bar, Fig 4C). We found that SARS‐CoV‐2 titer was similar between isotype control group (gray column, Fig 4D) and mice injected with CLEC2.Fc (blue columns, Fig 4D), suggesting CLEC2.Fc was unable to inhibit SARS‐CoV‐2 infection and replication. However, CLEC2.Fc efficiently inhibited the expression of proinflammatory cytokines (IL‐6, TNF‐α, IFN‐γ, and IL‐10) and chemokines (CXCL1, CXCL2, CXCL5, CCL2, IP‐10) at day 3 and day 5 post‐infection (blue columns, Fig 4E).

We further asked whether administration of CLEC2.Fc after SARS‐CoV‐2 inoculation was effective to reduce lung inflammation (therapeutic model, red bars). To address this question, mice were intraperitoneally (IP) injected with CLEC2.Fc (200 μg) at 8 h (lower red bar, Fig 4C) and day 3 (upper red bar, Fig 4C) after SARS‐CoV‐2 inoculation. Even though CLEC2.Fc was unable to inhibit virus infection and replication (Fig 4D), CLEC2.Fc inhibited the expression of IL‐6, CXCL2, CXCL5, CCL2, and IP‐10 at day 3 and day 5 post‐infection, and the expression of TNF‐α, IFN‐γ, IL‐10, and CXCL1 was also suppressed at day 5 post‐infection (red columns, Fig 4E). These observations demonstrate that CLEC2.Fc has potent anti‐inflammatory effects *in vivo*.

### CLEC2.Fc is able to inhibit SARS‐CoV‐2‐induced NET formation and collagen deposition

**Figure 5:**
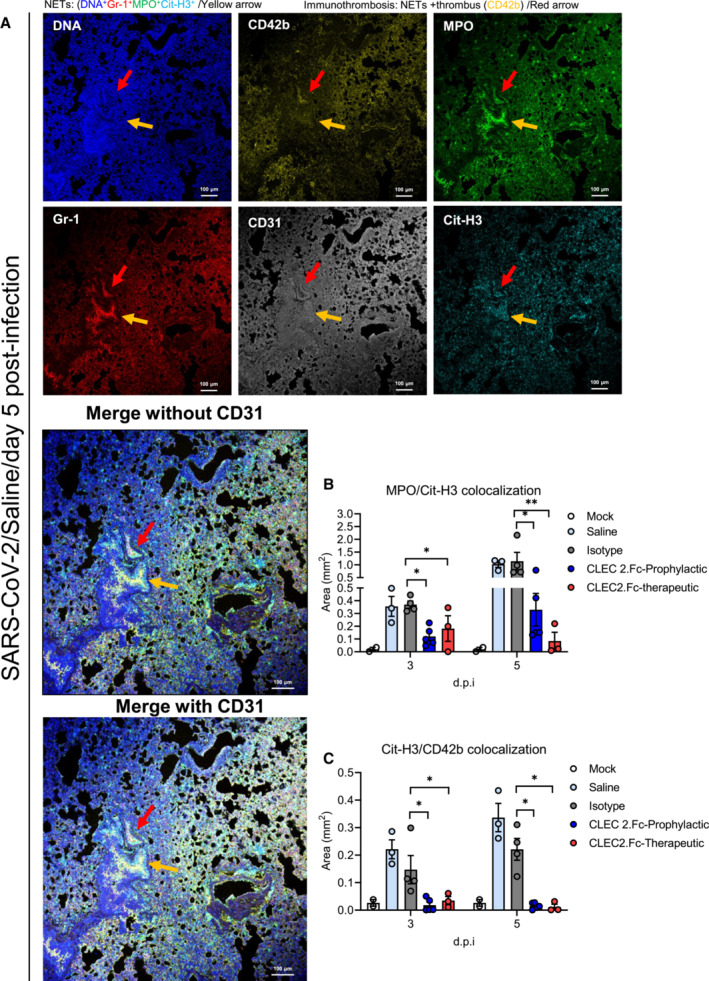
CLEC2.Fc inhibits SARS‐CoV‐2‐induced immunothrombosis in lung A
AAV‐hACE2 mice were treated with saline and challenged with SARS‐CoV‐2 (8 × 10^4^ PFU) for 5 days. Lung tissue sections were stained with DAPI (blue), anti‐MPO antibody (green), anti‐citrullinated histone H3 (Cit‐H3) antibody (red), anti‐CD42b antibody (yellow) and anti‐CD31 antibody (gray). yellow arrow: NETs (DNA^+^Gr‐1^+^MPO^+^Cit‐H3^+^); red arrow: immunothrombosis (NETs + thrombus (CD42b^+^)), Scale bar is 100 μm.B, C
For prophylactic treatment, CLEC2.Fc (or vehicle) was given at 1 h before virus challenge; for therapeutic treatment, CLEC2.Fc was given at 8 h post‐infection. The area of NET (colocalization area of MPO and Cit‐H3) (B) and immunothrombosis (colocalization area of Cit‐H3 and CD42b) (C) were measured using MetaMorph software. Data information: Data are mean ± SEM from two independent experiments (saline: *n* = 3 for 3 d.p.i., *n* = 4 for 5 d.p.i.; isotype: *n* = 3 for 3 d.p.i., *n* = 4 for 5 d.p.i.; prophylactic treatment of CLEC2.Fc treatment: *n* = 5 for 3 d.p.i. and 5 d.p.i.; therapeutic treatment of CLEC2.Fc: *n* = 3 for 3 d.p.i. and 5 d.p.i.). *P < 0.05, **P < 0.01 (two‐way ANOVA). Source data are available online for this figure.

We detected NET formation *in vivo* by examining DNA (Hoechst 33342), citrullinated histone H3 (Cit‐H3), and myeloperoxidase (MPO), as well as markers of neutrophils (Gr‐1), activated platelets (CD42b), and pulmonary endothelial cells (CD31) using the samples isolated from SARS‐CoV‐2‐infected mice in both prophylactic and therapeutic models. In the SARAS‐CoV‐2‐inoculated AAV‐ACE2 mice (Sun *et al*, 2021). Obvious NETs and immunothrombosis in lung (Fig 5) and heart (Appendix Fig S5A and B), but not in spleen (Appendix Fig S5C), were observed at day 5 post‐inoculation. While abundant NETs and CD42b‐associated thrombi were observed in lung (Fig 5A) and heart (Appendix Fig S5) of mice treated with saline (control group), CLEC2.Fc efficiently attenuated NET formation (quantitation of NET: MPO and Cit‐H3 colocalized area, Fig 5B) and immunothrombosis (quantitation of immunothrombosis: Cit‐H3 and CD42b colocalized area, Fig 5C) in both prophylactic (blue column) and therapeutic models (red columns). The images of CLEC2.Fc‐mediated suppressive effect on NET formation and immunothrombosis were shown in Appendix Fig S6.

**Figure 6:**
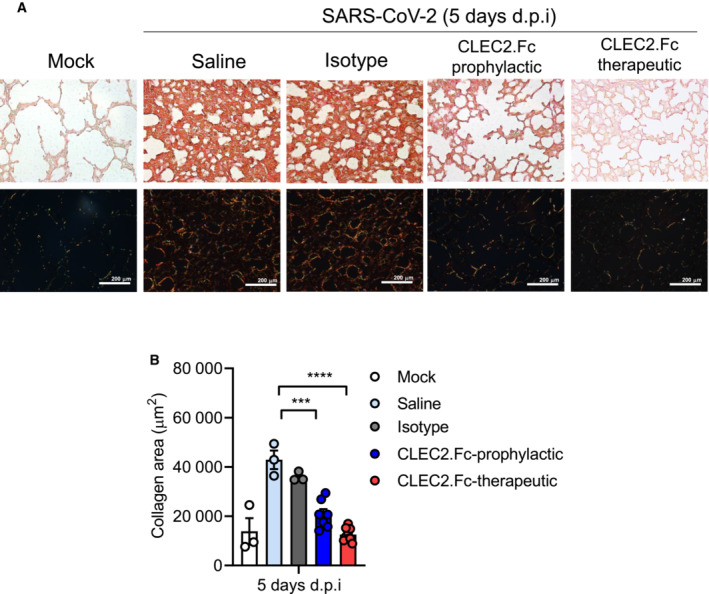
CLEC2.Fc inhibits SARS‐CoV‐2‐induced collagen deposition in lung A, B
AAV‐hACE2 mice were treated with saline and challenged with SARS‐CoV‐2 (8 × 10^4^ PFU) for 5 days. Samples were collected at day 5 post‐infection, and the collagen was stained with Picro Sirius red (A), while collagen deposition was quantified using MetaMorph software and presented as area (μm^2^) (B). The images were captured by light microscopy (upper panel) and polarized light microscopy (lower panel). Data information: Data are mean ± SEM from two independent experiments (saline: *n* = 3 for 3 d.p.i., *n* = 4 for 5 d.p.i.; isotype: *n* = 3 for 3 d.p.i., *n* = 4 for 5 d.p.i.; prophylactic treatment of CLEC2.Fc treatment: *n* = 5 for 3 d.p.i. and 5 d.p.i.; therapeutic treatment of CLEC2.Fc: *n* = 3 for 3 d.p.i. and 5 d.p.i.). ***P < 0.001, ****P < 0.0001 (two‐way ANOVA). Source data are available online for this figure.

In addition to NET formation, SARS‐CoV‐2 infection caused extensive cell infiltration, including interstitial macrophages, monocyte‐derived dendritic cells (DC)/macrophages (MΦ), and Ly6C^+^ monocytes (gray columns, Appendix Fig S7). In contrast, cell infiltration was inhibited by CLEC2.Fc in the prophylactic model (blue columns, Appendix Fig S7), but not in the therapeutic model (data not shown). Moreover, substantial collagen deposition (as revealed by Picro Sirius Red staining) in lungs was observed in SARS‐CoV‐2‐challenged mice at day 5 post‐infection in control group. In contrast, administration of CLEC2.Fc attenuated collagen expression efficiently in both prophylactic and therapeutic models (Fig 6A and B). These observations suggest that CLEC2.Fc is not only able to inhibit SARS‐CoV‐2‐induced thromboinflammation, but also attenuates collagen deposition *in vivo*.

To understand the pharmacokinetics (PK) and biodistribution of CLEC2.Fc, we injected the radioisotope‐labeled CLEC2.Fc and human IgG1 (hIgG1) [as isotype control] and detected the signals by single‐photon emission computed tomography (SPECT) and computed tomography (CT; Appendix Fig S8). Unlike the broad distribution of DTPA‐labeled hIgG1, DTPA‐CLEC2.Fc is rapidly cleared from blood and is mainly accumulated in liver up to 168 h (Appendix Fig S8A). The PK of ^111^In‐labeled CLEC2.Fc and hIgG1 was presented as radioactivity and protein concentration in blood (Appendix Fig S8B and C), and the half‐life for hIgG1 and CLEC2.Fc was 61.04 h (±7.45) and 35.15 h (±2.89), respectively. The biodistribution in specific organs was quantitated in Appendix Fig S8D and E.

We further examined the expression of *pdpn* mRNA and podoplanin before and after SARS‐CoV‐2‐inoculation. Compared to un‐infected mice, expression of *pdpn* mRNA was upregulated 1.5‐fold (Appendix Fig S9A). Moreover, we examined the expression of podoplanin by immunochemistry staining and found that podoplanin was upregulated after injection of CLEC2.Fc in both prophylactic and therapeutic model (Appendix Fig S9B). As human CLEC2.Fc also binds mouse podoplanin (Appendix Fig S10), this observation suggests that CLEC2.Fc may be recruited to lung after SARS‐CoV‐2 infection.

## Discussion

In this work, we demonstrate that CLEC2 plays critical roles in SARS‐CoV‐2‐induced thromboinflammation. As CLEC2 is expressed in platelets abundantly, and lung is the major extra‐bone marrow organ for platelet biogenesis and reservoir of megakaryocytes (Lefrancais *et al*, 2017; Lefrancais & Looney, 2019), SARS‐CoV‐2 is likely to induce severe immunothrombosis and cause acute respiratory syndrome in COVID‐19 patients via activating platelets in lung. Even though SARS‐CoV‐2 was reported to engage ACE2 to activate platelets and enhance thrombosis (Zhang *et al*, 2020), other groups were unable to confirm the presence of ACE2 in platelets (Manne *et al*, 2020; Song *et al*, 2020; Li *et al*, 2022). Instead, SARS‐CoV‐2 spike protein was reported to engage CD42b receptor to activate platelets, thereby promoted proinflammatory cytokine release from monocyte (Li *et al*, 2022). Because CLEC2 is a pattern recognition receptor in platelets (Sung *et al*, 2019), we are interested to understand whether CLEC2 contributes to SARS‐CoV‐2‐induced immunothrombosis and microemboli.

CLEC2 was shown to associate with DC‐SIGN to capture HIV to facilitate HIV dissemination in infected patients (Chaipan *et al*, 2006). Moreover, DV was shown to activate CLEC2 to release EVs from platelets (Sung *et al*, 2019), despite DV interacted with CLEC2 weakly (Chen *et al*, 2008). In contrast to HIV and DV, SARS‐CoV‐2 RBD bound CLEC2 and DC‐SIGN directly, suggesting SARS‐CoV‐2 is more potent than DV to activate platelets. This speculation is in accord with the induction of thread‐like and aggregated NETs by DV and SARS‐COV‐2, respectively. Unlike the thread‐like NETs, which adhered to glass plates, SARS‐COV‐2‐induced aggregated NETs detached from glass plate in the presence of platelets (Fig 3A and B, Appendix Fig S3). Even though it is still unclear why the SARS‐CoV‐2/platelet‐induced NETs are distinct from PMA and virus‐induced thread‐like NETs, our observation is in accord with the report that the levels of cell‐free DNA, MPO‐DNA complex, and citrullinated histone H3 (Cit‐H3) were elevated in blood samples of COVID‐19 patients (Zuo *et al*, 2020). Thus, we speculate that the detached NETs may form microemboli to cause microangiopathy in COVID‐19 patients.

We also demonstrated that CLEC2.Fc suppressed SARS‐CoV‐2‐induced thromboinflammation and collagen deposition (Fig 6). As collagen contributes to scar formation (Simoes *et al*, 2020), and NETosis can drive post‐COVID syndrome (Sawadogo *et al*, 2020; Zhu *et al*, 2022), CLEC2.Fc seems a promising therapeutic agent to protect host from SARS‐CoV‐2‐induced lung injury. The CLEC2.Fc‐mediated protective effect *in vivo* is not only limited to block SARS‐CoV‐2 RBD‐induced thromboinflammation, but may also attribute to its ability to inhibit the proinflammatory effects of podoplanin (CLEC2 endogenous ligand; Suzuki‐Inoue *et al*, 2006, 2010, 2011). Podoplanin has been identified as an alveolar type I epithelial cell‐specific antigen in lung (Dobbs *et al*, 2010), and podoplanin‐positive macrophages are able to activate platelet and induce aggregation during inflammatory reactions (Kerrigan *et al*, 2012). Furthermore, podoplanin‐CLEC2 interaction contributes to venous thrombosis (Suzuki‐Inoue, 2017), suggesting blockade of podoplanin‐mediated inflammation by CLEC2.Fc may be able to reduce the risk of vascular microthrombosis in COVID‐19 patients. Therefore, CLEC2.Fc can complement the neutralization effect of anti‐spike RBD antibody, and combination of CLEC2.Fc and anti‐spike RBD antibody may further decrease the risk of developing long COVID‐19 syndromes.

As persistent circulating SARS‐CoV‐2 spike protein is detected in COVID‐19 patients' blood samples and is associated with post‐acute sequelae of COVID (Swank *et al*, 2023), CLEC2.Fc may bind the circulating SARS‐CoV‐2 spike protein to prevent the activation of platelets and alveolar macrophages, thereby attenuates SARS‐CoV‐2‐induced thromboinflammation in lung. In addition, podoplanin is upregulated in infected lung tissue (Appendix Fig S9); thus, CLEC2.Fc could also attenuate lung inflammation via blocking podoplanin‐platelet interaction.

Even though both SARS‐CoV RBD (Li *et al*, 2003; Wang *et al*, 2004) and SARS‐CoV‐2 RBD (Hoffmann *et al*, 2020; Yan *et al*, 2020) can bind ACE2, it is interesting to find that only SARS‐CoV‐2 RBD can bind CLEC2 (Fig 2). This observation suggests that variation of amino acid residues in SARS‐CoV RBD contributes to the loss of CLEC2‐binding ability, and this can explain why severe immunothrombosis is reported in COVID‐19 patients, but is rarely found in SARS‐CoV‐infected victims. It will be very interesting to test whether engineered SARS‐CoV‐2 RBD, with attenuated binding ability to CLEC2, can reduce platelet activation and still elicit protective immunity against SARS‐CoV‐2 invasion. While CLEC2.Fc has shown efficacy in reducing SARS‐CoV‐2‐induced immunothrombosis, its low affinity for Spike.RBD raises concerns of potential off‐target binding *in vivo*. Therefore, it is essential to develop a CLEC2 mutant with a higher binding affinity for both Spike.RBD and podoplanin. Alternatively, a small molecule inhibitor that selectively blocks CLEC2 activation could be a promising approach to inhibit the interaction between the pathogen and CLEC2 and prevent immunothrombosis in the future.

We have fused the ligand‐binding domains of Syk‐coupled C‐type lectin receptors and CD33‐related SIGLECs to capture various viruses. We find that Syk‐coupled CLEC5A and the immunoreceptor tyrosine‐based inhibitory motif (ITIM)‐containing SIGLEC‐3 is the pattern recognition receptors for flaviviruses (Chen *et al*, 2008, 2012; Wang *et al*, 2023) and hepatitis B virus (Tsai *et al*, 2021), respectively. In this study, we further find that CLEC2 is the pattern recognition receptor for SARS‐CoV‐2, suggesting C‐type lectins and SIGLECs are critical in virus‐induced inflammatory reactions (Saini *et al*, 2022). Recently, several members of C‐type lectins have been shown to exacerbate proinflammatory responses in SARS‐CoV‐2 infections, including DC‐SIGN, L‐SIGN, LSECtin, ASGR1, and CLEC10A (Lu *et al*, 2021). Even though these C‐type lectins are not involved in virus replications, engagement of SARS‐CoV‐2 spike protein with myeloid cells induces proinflammatory cytokines (Lu *et al*, 2021). In this work, we demonstrated that SARS‐CoV‐2 RBD spike engaged Syk‐coupled CLEC2 to activate platelets, thereby contributed to thromboinflammation in COVID‐19 patients (Sung & Hsieh, 2021). These observations further demonstrate the critical roles of C‐type lectin and platelets in viral infections, and targeting C‐type lectins may be a novel strategy to inhibit thromboinflammation in the future.

## Materials and Methods

### Reagents and antibodies

All culture media and buffers were purchased from Gibco. Blocking assays, using anti‐human CLEC2 mAb (clone AYP1), were as previously described^18^. Antibodies for immunofluorescence staining and IHC include rabbit anti‐citrullinated Histone H3/Cit‐H3 antibody (#NB100‐57135, Novus; dilution 1:100), goat anti‐human/mouse myeloperoxidase (MPO) Polyclonal Antibody (# AF3667, R&D systems; dilution 1:100), rabbit anti‐CD11b antibody (#ab13357, Abcam; dilution 1:100), rabbit anti‐CD64 antibody (#MA5‐29704, Invitrogen; dilution 1:100), rabbit anti‐F4/80 antibody (#ab74383, Abcam; dilution 1:100), mouse anti‐CCR2 antibody (#NBP‐35334, Novus Biologicals; dilution 1:100), goat anti‐Ly6C antibody (#SC‐23080, Santa Cruz; dilution 1:100), rabbit anti‐CD31 antibody (#GTX130274, Genetex; dilution 1:100), rat anti‐Gr‐1 antibody (#ab25377, Abcam; dilution 1:100), goat anti‐mouse CD42b antibody (#IM0409; dilution 1:100). Secondary antibodies included donkey anti‐rabbit IgG (H + L) Alexa488‐conjugated antibody (#715‐545‐152, Jackson ImmunoResearch), donkey anti‐goat IgG (H + L) Alexa647‐conjugated antibody (#705‐605‐147, Jackson ImmunoResearch), HRP‐conjugated donkey anti‐human IgG (H + L; #709‐035‐149, Jackson ImmunoResearch), HRP‐conjugated donkey anti‐rabbit IgG (H + L; #711‐035‐152, Jackson ImmunoResearch), HRP‐conjugated bovine anti‐goat IgG (H + L; #805‐035‐180, Jackson ImmunoResearch), HRP‐conjugated donkey anti‐mouse IgG (H + L; #715‐035‐150, Jackson ImmunoResearch), HRP‐conjugated goat anti‐rat antibody (#112‐035‐143, Jackson ImmunoResearch), and all of the secondary antibodies were used in 100 times dilution. For ELISA binding assay, anti‐SARS‐CoV‐2 S2 antibody (# GTX632604, GeneTex; 5 μg/ml) and anti‐SARS‐CoV‐2 S1 recombinant antibody (# 938702, BioLegend; 45 ng/ml) were used according to vendors' instructions. SARS‐CoV‐2 spike RBD proteins (wild type/WT, alpha, beta, gamma, delta, omicron BA.1, omicron BA.2, omicron BA.3, omicron BA.4 &BA.5) of SARS‐CoV‐2 spike RBD protein and SARS‐CoV spike RBD were purchased from Acro Biosystem. The wild‐type trimeric SARS‐CoV‐2 spike RBD is also purchased from Acro Biosystem.

### Determination of C‐type lectin and SARS‐CoV‐2 RBD interaction by ELISA

The SARS‐CoV‐2 RBD (5 μg/ml in PBS; 50 μl/well) was coated in a 96‐well microtitration plates (Costar #9018) and stored at 4°C overnight. Plates were washed once with PBST (PBS containing 0.05% Tween20) using a HydroFlex™ microplate washer and then blocked with 100 μl of 3% BSA in PBST for 1 h at room temperature. After washing, 50 μl of various recombinant human C‐type lectin.Fc fusion proteins (50 μg/ml, 1% BSA in PBS; 50 μl/well), healthy control (HC, unvaccinated with SARS‐CoV‐2 vaccine) serum (dilution 1:1,000), COVID‐19 patient's serum (dilution 1:1,000), or anti‐SARS‐CoV‐2 S2 antibody (GeneTex # GTX632604, 5 μg/ml), or anti‐SARS‐CoV‐2 S1 Recombinant Antibody (BioLegend # 938702, 45 ng/ml) were added and incubated for 2 h at room temperature. Plates were washed with PBST three times and incubated with 50 μl of HRP‐conjugated anti‐human IgG (H + L) antibody (dilution 1:10,000) for 1 h at room temperature. HRP activity was revealed by the addition of 100 μl TMB substrate (3,3′,5,5′‐tetramethylbenzidine) for 15 min, and then, the reaction was stopped by 50 μl of 2 N H_2_SO_4._ The optical density at 450 nm was measured by an microplate reader (Sunrise, TECAN).

### Measurement of binding affinity between C‐type lectin.Fc fusion proteins and SARS‐CoV‐2 RBD

The construction CLEC2.Fc cDNA and expression of CLEC2.Fc were as described previously (Chen *et al*, 2008; Sung *et al*, 2019). In brief, the extracellular domain of human CLEC2 was amplified by RT–PCR, then fused with the Fc portion of human IgG1 before transfected into 293T cells to generate CLEC2.Fc fusion protein. The map of CLEC2.Fc construct is shown in Appendix Fig S11. The interactions between C‐type lectin‐Fc fusion proteins and SARS‐CoV‐2 RBD were determined by the Bio‐layer interferometry (BLI, ForteBio). In brief, anti‐Penta‐His (HIS1K) tips (Forte ´Bio) were soaked in PBS buffer and shaken on Sidekick Shaker (Forte ´Bio) for 20 min before they were used for the binding assay in an Octet HTX (Forte ´Bio). All steps were performed at 30°C with an agitation speed of 1,000 rpm on the flat bottom 96‐well black plate (Greiner bio‐one). COVID‐2019‐Spike‐328‐585 (RBD) was immobilized at 20 μg/ml in PBS to HIS1K tips for 600 s to a response level of around 0.8 nm. Biosensor tips were then equilibrated for 60 s in PBS buffer before assessment of binding for 240 s followed by dissociation for 240 s. Human IgG1 and recombinant CLEC2.Fc, DC‐SIGN.Fc and IgG1 were serially diluted two‐fold in PBS (50 μM, 31 μM, and 8.8 μM). Biosensors were regenerated by three 5 s exposures to regeneration buffer (10 mM glycine pH 2.0, Amresco) between each assay. Data analysis and curve fitting (1:1 binding model) was carried out with the Octet Data Analysis software v9.0.0.10 (Forte ´Bio). The measurement was performed in triplicate and shown as mean ± SD.

### Isolation of human neutrophils and platelets

Whole blood was collected from drug‐free healthy donors for the isolation of neutrophils and platelets as described previously (Koupenova & Freedman, 2020). In brief, fresh blood was mixed with anticoagulant ACD (ratio 1:6, v/v), followed by centrifugation at 230 *g* for 15 min to collect platelet‐rich plasma (PRP). Platelets were harvested by further centrifugation at 1,000 *g* for 10 min, and the pellet was suspended in Tyrode's buffer. For the isolation of neutrophils, blood was laid on Ficoll‐Paque (GE Healthcare, 45‐001‐748) then centrifuged at 500 *g* for 15 min. Red blood cells (RBCs) were lysed with RBC lysis buffer and washed with saline. Neutrophils were suspended in RPMI containing 10% autologous serum. This study was conducted under the Helsinki Declaration of 1975. All patients provided written informed consent before enrollment, and the study was approved by the Human Subject Research Ethics, Academia Sinica (AS‐IRB‐BM‐20025).

### Isolation of murine neutrophils and platelets

Mouse platelets were isolated from peripheral blood and neutrophils were isolated from bone marrow; Percoll gradients were used to separate the neutrophils as previously described (Sung *et al*, 2019).

### Production and purification of pseudotyped lentivirus

The pseudotyped lentivirus carrying the SARS‐Co‐2 spike protein was generated as described previously (Glowacka *et al*, 2011). In brief, HEK‐293T cells were transiently transfected with pLAS2w.Fluc.Ppuro, pcDNA3.1‐2019‐nCoV‐S, and pCMV‐▵R8.91 using TransITR‐LT1 transfection reagent (Mirus). The culture medium was refreshed at 16 h and harvested at 48 h and 72 h post‐transfection by Bio‐layer interferometry. Cell debris was removed by centrifugation at 4,000 *g* for 10 min, and the supernatant was passed through 0.45 μm syringe filter (Pall Corporation). For pseudotyped virus purification and concentration, the supernatant was mixed with 0.2× volume of 50% PEG 8000 (Sigma) and incubated at 4°C for 2 h. The pseudotyped lentivirus was then recovered by centrifugation at 5,000 *g* for 2 h and resolved in sterilized phosphate‐buffered saline, aliquoted, and stored at −80°C. All the pseudotyped lentivirus‐related experiments were performed in Biological Safety level 2 (BSL2) laboratory.

### Mouse model for SARS‐CoV‐2 infection

Virus preparation and inoculation of SARS‐CoV‐2 into C57BL/6 mice were as described (Sun *et al*, 2021). All the C56BL/6 J used in the animal experiments were provided by National Laboratory Animal Center (NLAC), NARLabs, Taiwan. In brief, mice were infected with recombinant AAV‐hACE2 by intra‐tracheal route for two weeks, followed by inoculation of SARS‐CoV‐2 for seven days before sacrifice. For prophylactic treatment, AAV‐hACE2 mice were intravenously injected with hCLEC2.Fc (100 μg/per mice) at 1 h before SARS‐CoV‐2 inoculation; the second dose of CLEC2.Fc (100 μg/per mice) was administered intraperitoneally at day 3 post‐infection. For therapeutic treatment, mice were intraperitoneally injected with CLEC2.Fc (200 μg/per mice) at 8 h after SARS‐CoV‐2 inoculation and again at day 3 post‐infection. Lung tissue was collected at day 3 and day 5 post‐infection for further analysis. All the animal experiments with SARS‐CoV‐2 were performed in a Biological safety level 3 (BSL3) laboratory and with approval from the Institutional Animal Care and Use Committee (IACUC) at AS core (protocol ID 20‐10‐1521).

### Induction of neutrophil extracellular traps (NETs)

Neutrophils (4 × 10^5^/ml) were stimulated with SARS‐CoV‐2 (MOI = 0.1 or 1) and co‐incubated with autologous platelets (4 × 10^6^/ml) for 5 or 20 h at 37°C. For blocking assays, platelets were preincubated with anti‐CLEC2 mAb (2.5 μg/ml) or isotype control (2.5 μg/ml) for 15 min at room temperature then co‐cultured with neutrophils on poly‐L‐lysine‐coating glass coverslips and stimulated with virus. For the CLEC2.Fc competition assay, human neutrophils were incubated with SARS‐CoV‐2 (MOI = 0.1 or 1) in the presence of hCLEC2.Fc (10 μg/ml) or anti‐RBD neutralizing antibody (10 μg/ml, a gift from Dr. Han‐Chung Wu, ICOB, Academia Sinica) for 20 h at 37°C. For the murine cell stimulation, neutrophils (4 × 10^5^/ml) from WT mice were stimulated with SARS‐CoV‐2 (MOI = 1) and co‐incubated with platelets (4 × 10^6^) from WT mice or *clec2* ^*−/−*^ mice for 5 h at 37°C.

### Visualization and quantification of NET structure

Samples were fixed with 4% paraformaldehyde and then permeabilized with 0.5% Triton X100 in PBS for 15 min. Components of NETs were visualized by staining with anti‐MPO antibody (2 μg/ml), anti‐citrullinated histone antibody (3 μg/ml), and Hoechst 33342 (0.5 μg/ml). The histone area of NETs was determined from images captured using a Leica confocal microscope with white light laser system (TCS SP8 X‐FALCON) and analyzed using MetaMorph™ software.

### Multiplex fluorescent staining of paraffin‐embedded tissue and measurement of NET/Immunothrombosis

Samples were fixed in 10% formalin for 72 h and then embedded in paraffin. Tissue sections were deparaffined and rehydrated then stained with primary antibodies (rabbit anti‐citrullinated Histone H3/Cit‐H3 antibody, #NB100‐57135, Novus; dilution 1:100) at 4°C overnight. After incubation with secondary antibodies (anti‐rabbit IgG (H + L) Alexa488‐conjugated antibody, Jackson ImmunoResearch; dilution 1:100), fluorophores were added as instructed in the Opal™ 7‐Color IHC Kit manual (Akoya Biosciences #NEL811001KT). Images were captured using a Leica confocal microscope with white light laser system (TCS SP8 X‐FALCON) and analyzed using MetaMorph™ software. Whole lung was scanned to measure the colocalization area of Cit‐H3 and MPO (as NET formation) as well as the colocalization area of Cit‐H3 and CD42b (immunothrombosis). The primary antibodies are rabbit anti‐citrullinated Histone H3/Cit‐H3 antibody (#NB100‐57135, Novus; dilution 1:100) and goat anti‐human/mouse myeloperoxidase (MPO) polyclonal antibody (# AF3667, R&D systems; dilution 1:100). Secondary antibodies are donkey anti‐rabbit IgG (H + L) Alexa488‐conjugated antibody (#715‐545‐152, Jackson ImmunoResearch; dilution 1:100) and donkey anti‐goat IgG (H + L) Alexa647‐conjugated antibody (#705‐605‐147, Jackson ImmunoResearch; dilution 1:100).

### Detection of MPO‐elastase‐DNA complex

Human neutrophils (4 × 10^5^/100 μl) were incubated with SARS‐CoV2‐spike Pseudotyped virus (WT, MOI = 0.1) in present autologous platelets (4 × 10^6^/100 μl) for 3 h at 37°C. Supernatant was harvested by centrifugation at 500 *g* for 5 min at room temperature. 96‐well Clear Flat Bottom Polystyrene High Bind Microplate (#9018, Corning) was coated with 50 μl of rabbit anti‐MPO serum (#07‐496, Millipore; dilution 1:1,000) and rabbit anti‐elastase polyclonal antibody (# sc‐25621, Santa Cruz; clone H‐57; 2 μg/ml) at 4°C overnight (14‐16 h). Plate was washed twice with PBS and then incubated with 50 μl of sample for 2 h at room temperature. After washing three times with 200 μl PBST (PBS containing 0.05% Tween 20), add 100 μl of detection antibody (HRP‐conjugated anti‐DNA antibody from Roche #Cell Death Detection ELISA; dilution 1:40) then incubated for 1 h at room temperature. Plate was washed three times with 200 μl PBST and then incubated with 200 μl of ABTS for 10–20 min. Measure the absorbance at 405/490 nm.

### Collagen staining and quantification

SARS‐CoV‐2‐challenged mice were sacrificed at day 5 post‐infection and tissues were fixed in 10% formalin for 72 h and then embedded in paraffin. Samples were deparaffined and rehydrated to stain with Picro Sirius Red (Abcam) for the visualization of collagen deposition. Images were captured by light microscopy and polarized microscopy under 40 × magnification. The level of collagen deposition was measured using MetaMorph™ software and presented as area (μm^2^).

### Isolation of RNA and real‐time qPCR

Tissues were homogenized in 700 μl of TRIzol reagent and RNA was isolated according to the vendor's instruction. RNA was transcribed to cDNA by reverse transcriptase PCR and subjected to real‐time qPCR as follows: incubation at 95°C for 5 min, followed by 30 cycles of 15 s at 95°C, 30 s at 58°C, and 30 s at 72°C. The expression level of each gene was normalized to GADPH expression. Primer sequences are listed in Appendix Table S2.

### Preparation of DTPA‐hCLEC2 fc and DTPA‐hIgG1

The CLEC2.Fc and hIgG1 were conjugated with 25‐fold molar excess of diethylenetriaminepentaacetic acid (DTPA) dianhydride (Sigma‐Aldrich, Missouri, USA) in 0.1 M bicarbonate buffer (pH 8.5) at room temperature for 2 h. The DTPA‐conjugated CLEC2.Fc and hIgG1 were purified using a Vivaspin® 500 concentrator (10 K MWCO, Sartorius, Goettingen, Germany). The DTPA‐conjugated CLEC2.Fc and hIgG1 were washed thrice with PBS to remove the unconjugated DTPA by centrifugation at 12,000 *g* for 10–20 min at 4°C.

### Preparation of radioisotope‐labeled CLEC2.Fc and hIgG1

For ^111^In‐labeling, DTPA‐conjugated CLEC2.Fc (1 mg) and DTPA‐conjugated hIgG1 (1 mg) was suspended in 200 μl of sodium acetate buffer (0.1 M, pH = 6.8), 8 mCi of 111InCl3 (INER, Taoyuan, Taiwan), respectively, and incubated at 37°C for 60 min. The mixtures were further purified by using Vivaspin® 500 concentrator (10 K MWCO, Sartorius, Goettingen, Germany) to remove the unlabeled ^111^In as mentioned above. Finally, the concentrated and purified samples were responded in PBS. The specific activity of ^111^In‐DTPA‐CLEC2.Fc and ^111^In‐DTPA‐hIgG1 were > 4 mCi/mg, respectively. The radiochemical purities of ^111^In‐DTPA‐CLEC2.Fc and ^111^In‐DTPA‐hIgG1 were measured using instant thin layer chromatography (iTLC, Agilent Technologies, Santa Clara, CA); 100 mM EDTA in 100 mM ammonium acetate acts as a mobile phase (^111^In‐labeled compounds Rf = 0, ^111^In‐EDTA Rf = 1). The iTLC sheets were analyzed using a radioactive scanner (AR‐2000 radio‐TLC Imaging Scanner, Bioscan, France). The radiochemical purity > 90% was acceptable for the consequent use in animal studies.

### Pharmacokinetic analysis (PK) and biodistribution of CLEC2.Fc

For pharmacokinetic analysis, mice were intravenously injected with 1 mCi of ^111^In‐DTPA‐CLEC2.Fc and ^111^In‐hIgG1 (equivalent to 10 mg/kg of protein; *n* = 3), respectively. The blood samples were collected at 0.5, 1, 2, 4, 24, 48, 96, and 168 h post‐injection, and mice were sacrificed by cervical vertebra dislocation at 168 h post‐administration. Blood samples and organs harvested to measure the uptake of radioactivity by a gamma counter (PerkinElmer, Waltham, MA, USA). Data are expressed as the percentage of injected dose per gram of organ (%ID/g).

### SPECT/CT imaging

The NanoSPECT/CT (Mediso Medical Imaging Systems, USA) plus scanner system was used to detect the ^111^In‐DTPA‐proteins *in vivo*. After administration of ^111^In‐DTPA‐CLEC2.Fc or ^111^In‐DTPA‐hIgG1, mice were inhaled anesthetic of 1–2% isoflurane during the imaging acquisition. SPECT and X‐ray CT images were obtained at 2, 24, 48, 96, and 168 h after injection. NanoSPECT imaging was acquired using nine multipinhole gamma detectors and high‐resolution collimators. The energy window was set to 171 and 245 KeV ± 10%, the image size was set to 256 × 256, and the field of view of 60 mm × 100 mm.

### Statistical analysis

All statistical analyses were performed by GraphPad Prism software (version 9.0), and the data were shown as dot plot with bar charts. Ordinary two‐way ANOVA was used in multiple‐groups comparison. For two‐groups comparison, nonparametric unpaired Student *t*‐test with Mann–Whitney test was used.

## Author contributions

**Pei‐Shan Sung:** Conceptualization; data curation; formal analysis; validation; investigation; visualization; methodology; writing – original draft; writing – review and editing. **Cheng‐Pu Sun:** Methodology. **Mi‐Hua Tao:** Methodology. **Shie‐Liang Hsieh:** Conceptualization; supervision; funding acquisition; writing – review and editing.

## Disclosure and competing interests statement

The authors declare that they have no conflict of interest.

## Supporting information



Appendix

Source Data for Appendix

Source Data for Figure 1

Source Data for Figure 3

Source Data for Figure 4

Source Data for Figure 5

Source Data for Figure 6

## Data Availability

This study includes no data deposited in external repositories.
