# Supplementary material for: Inhibition of SARS‐CoV‐2‐mediated thromboinflammation by CLEC2.Fc
Source: EMBO Mol Med. 2023 May 22;15(7):e16351. doi: 10.15252/emmm.202216351 (PMC10331576; doi:10.15252/emmm.202216351)
Supplement: Supplementary file 1 — Appendix [file EMMM-15-e16351-s003.pdf]

Table of contents to the appendix file

Contents	Page
Appendix Figure S1	1
Appendix Figure S2	2
Appendix Figure S3	3
Appendix Figure S4	4
Appendix Figure S5	5
Appendix Figure S6	6
Appendix Figure S7	7
Appendix Figure S8	8
Appendix Figure S9	9
Appendix Figure S10	10
Appendix Figure S11	11
Appendix figure legends	12-15
Appendix Table S1	16
Appendix Table S2	17

Appendix Figure S1

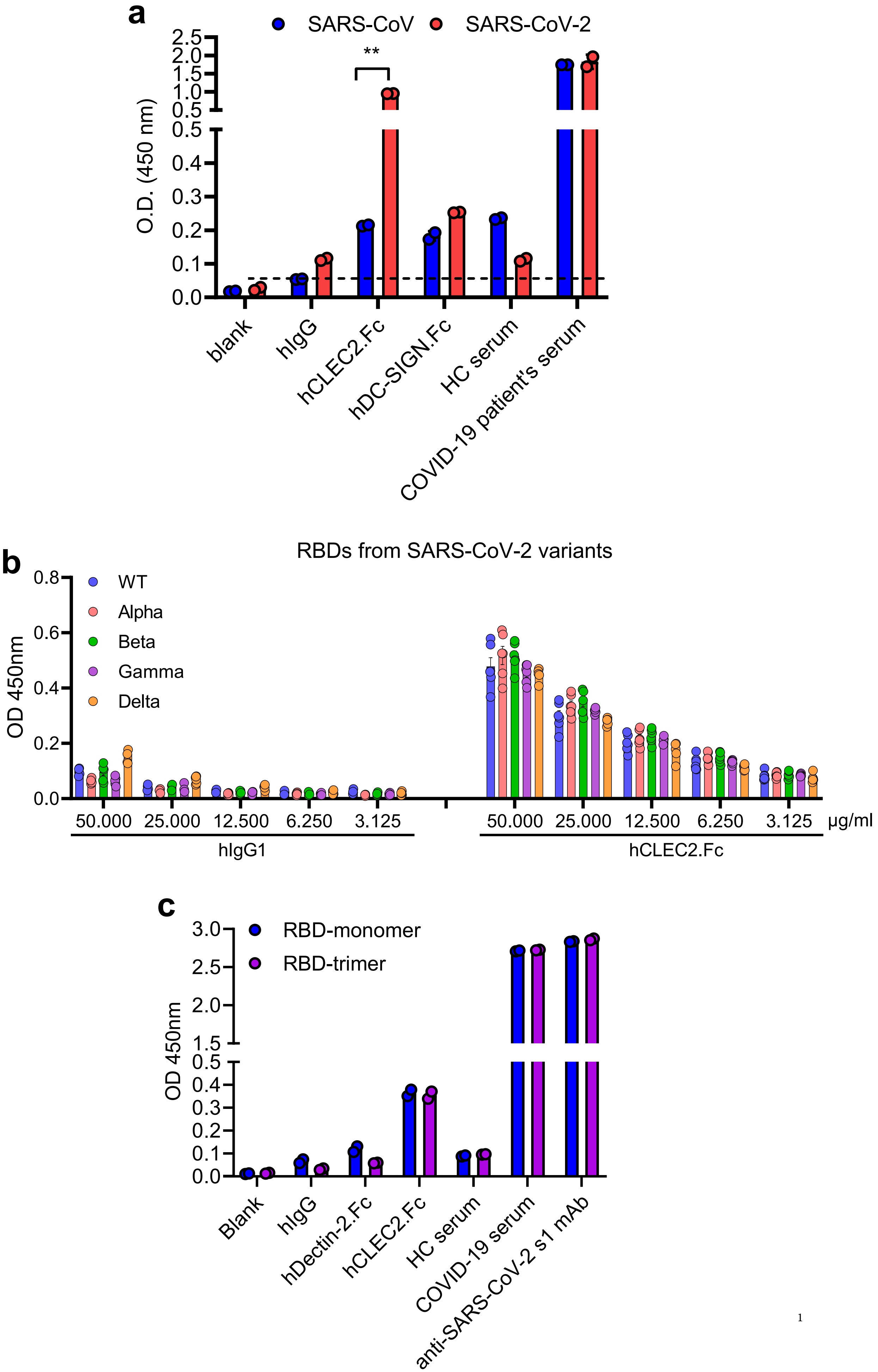

Appendix Figure S2

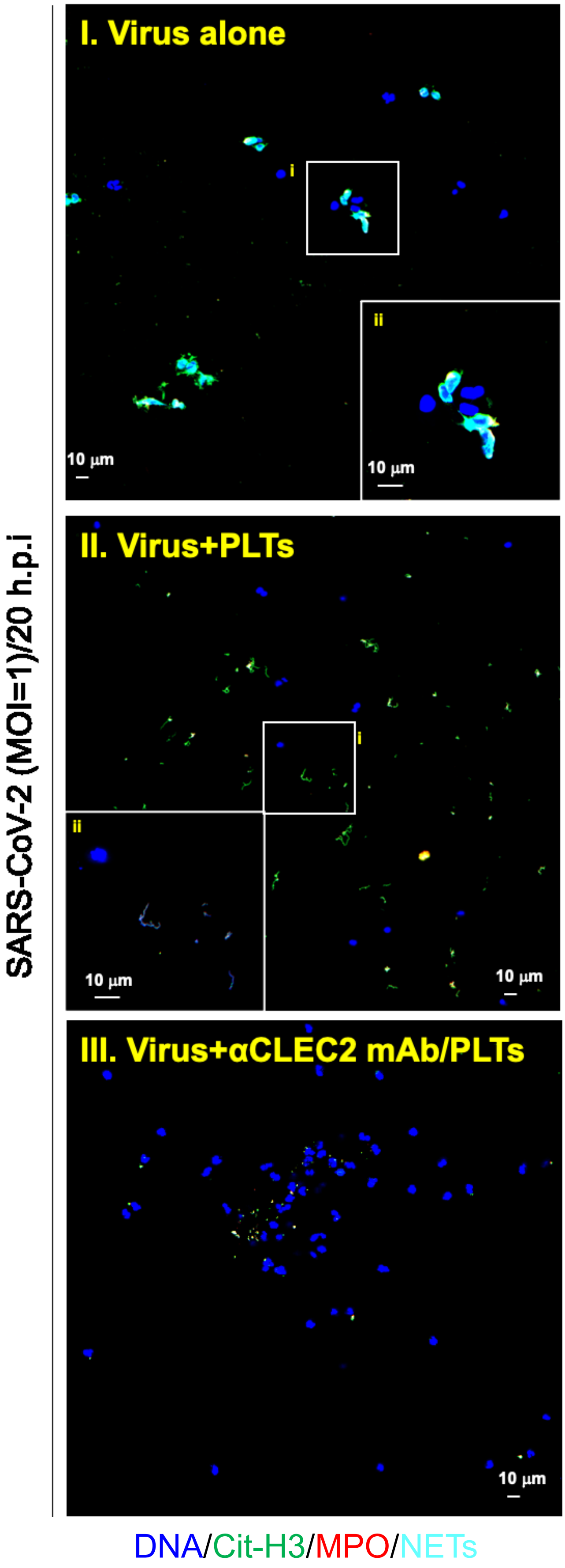

Appendix Figure S3

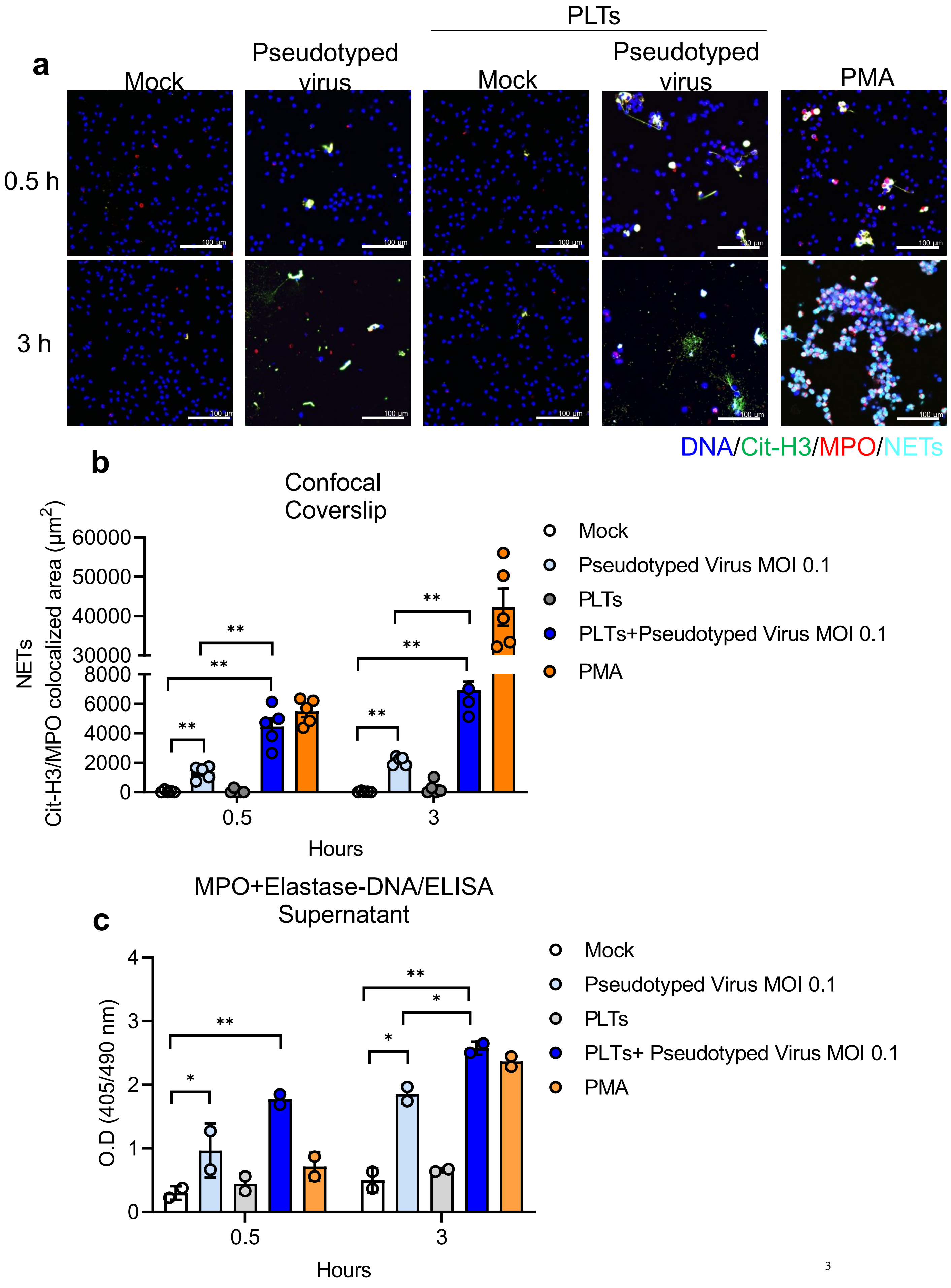

Appendix Figure S4

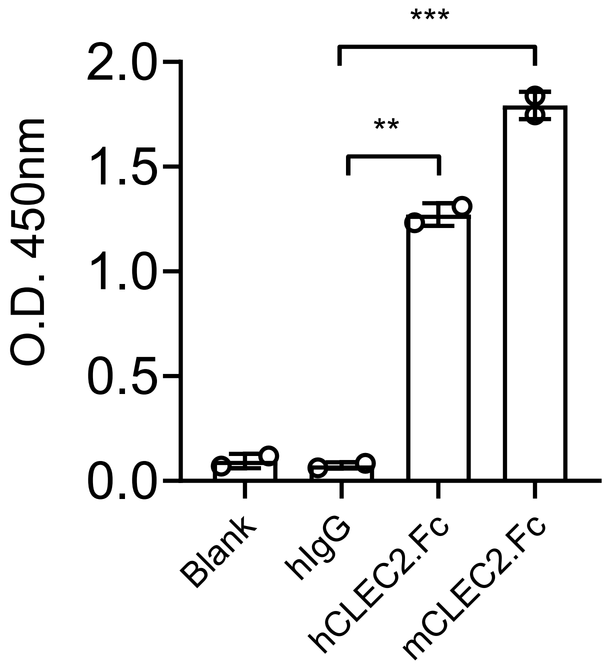

Appendix Figure S5

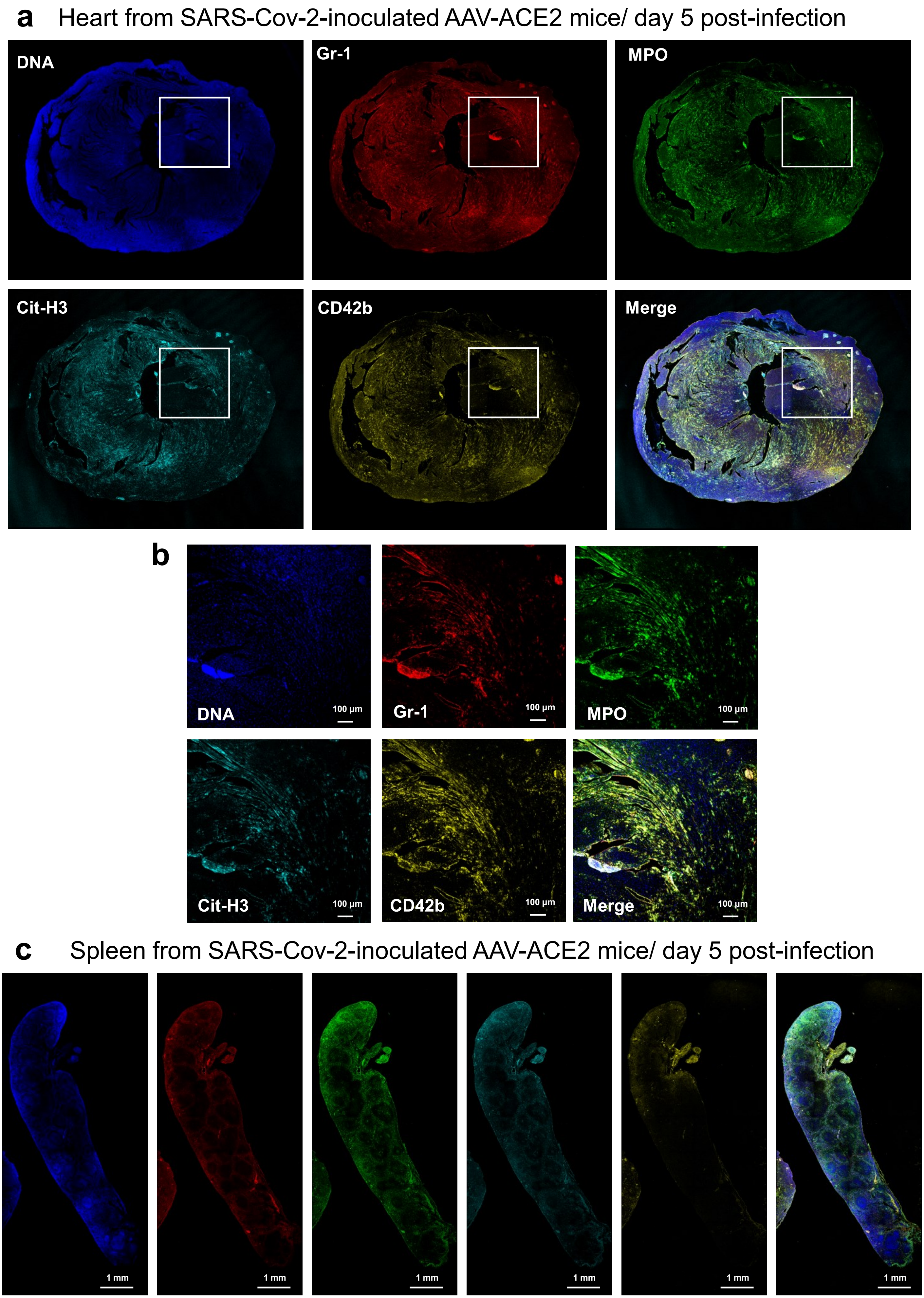

Supplementary figure 6

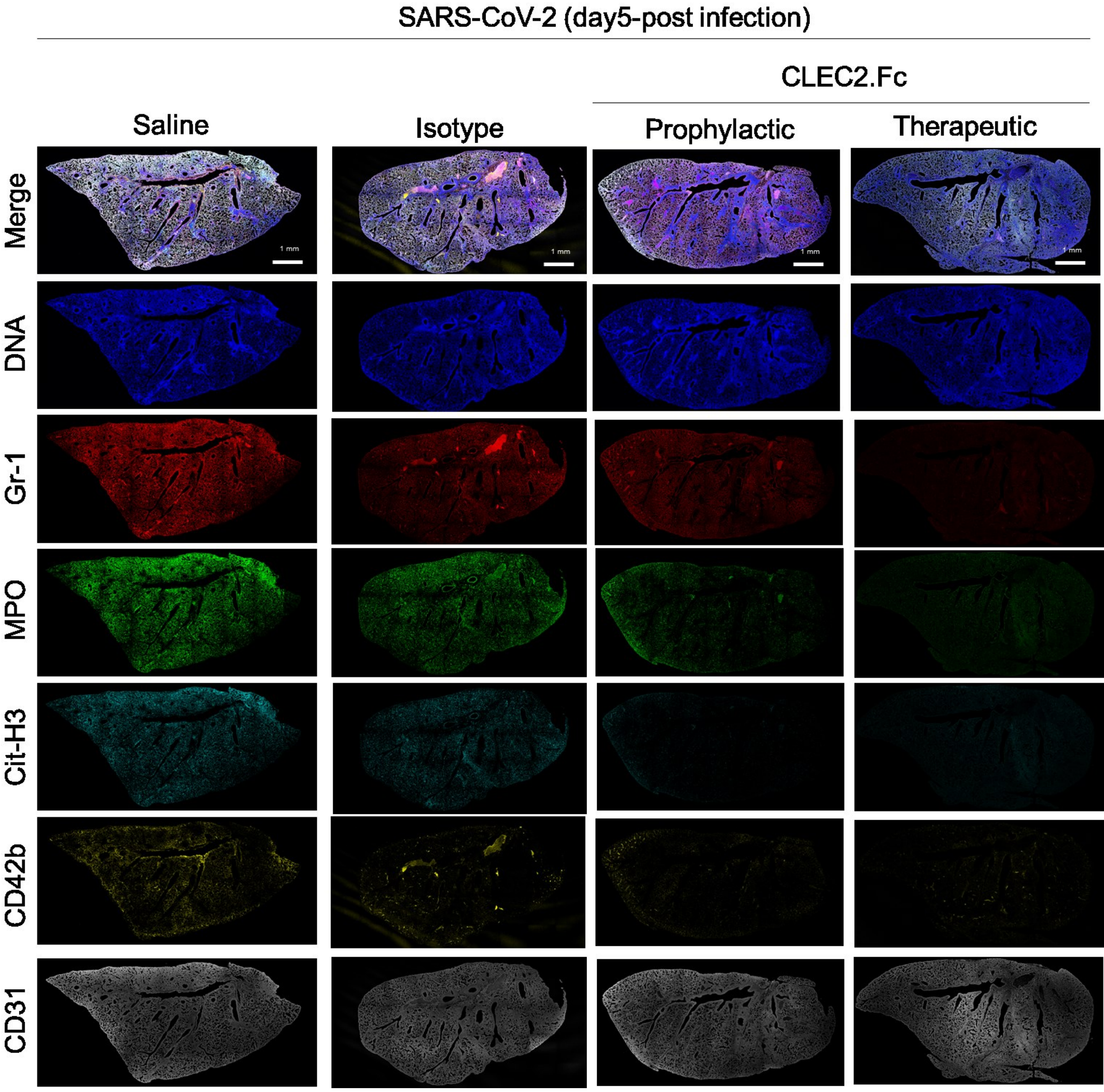

Appendix Figure S7

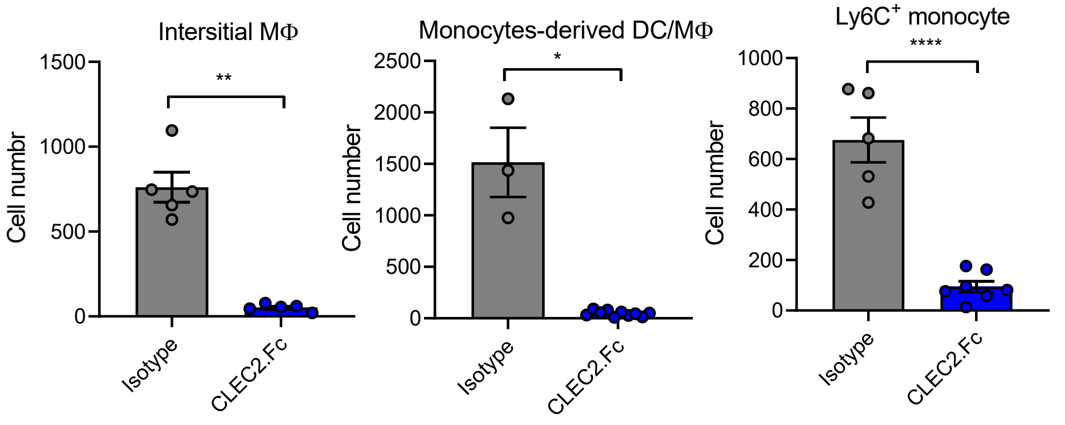

Appendix Figure S8

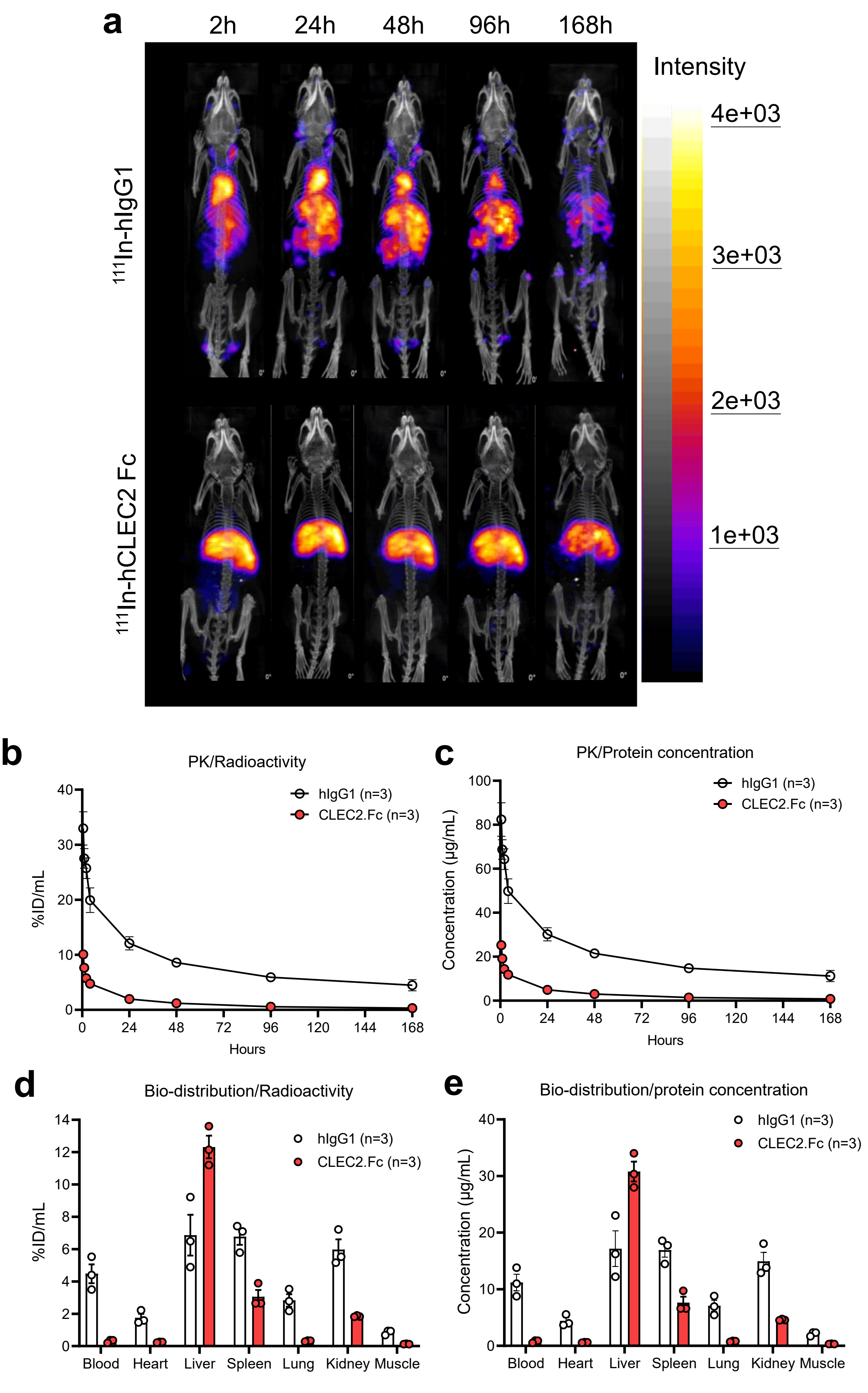

Appendix Figure S9

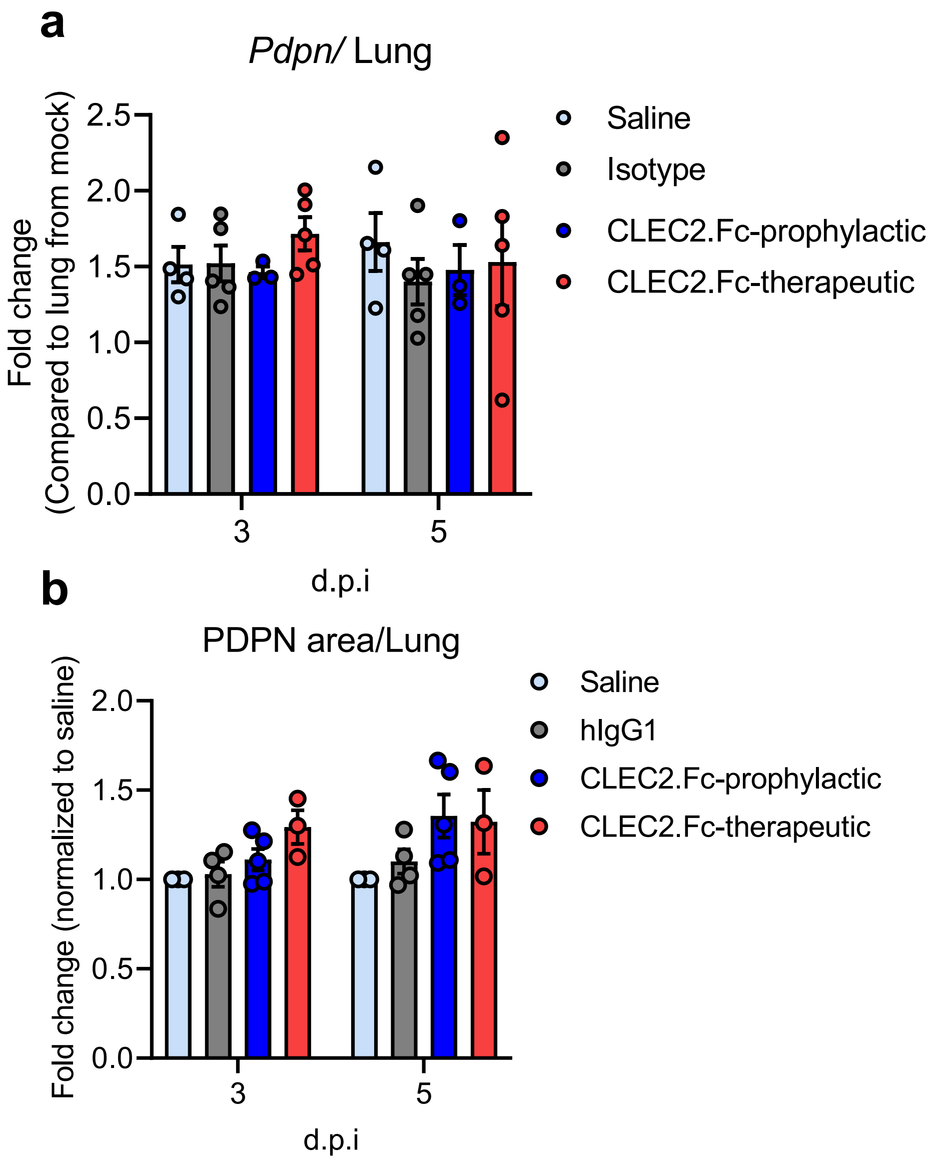

Appendix Figure S10

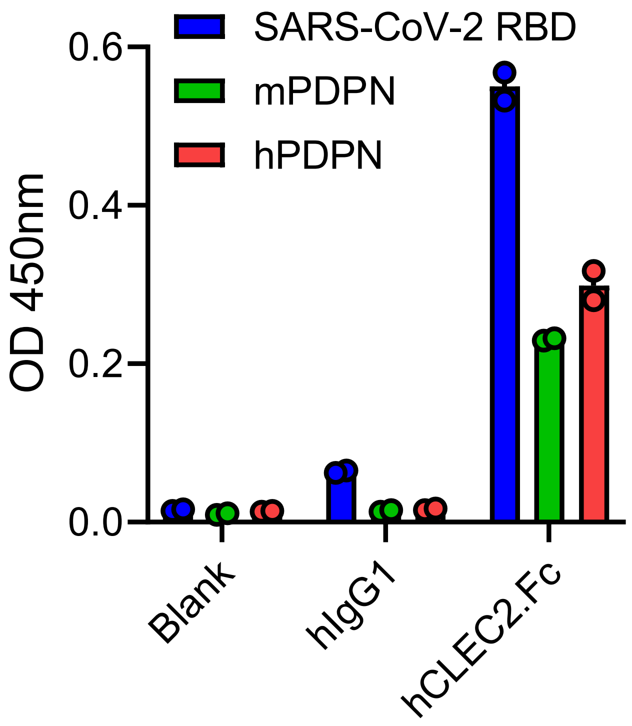

Appendix Figure S11

a

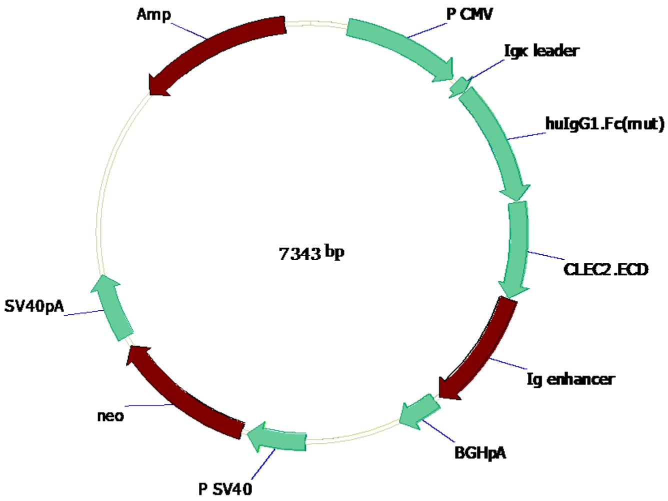

pcDNA3/huIgG1.Fc(mut)-huCLEC2.ECD

Appendix figure legends

Appendix Figure S1. Interactions between C-type lectins and RBDs of SARS-CoV-2 and SARS-CoV. (a) Comparison of human CLEC2.Fc (hCLEC2.Fc) and DC-SIGN (hDC-SIGN.Fc) binding to SARS-CoV spike RBD (blue column) and SARS-CoV-2 spike RBD (red column) by ELISA. (b) Serial dilution of hCLEC2.Fc and human IgG1 (hIgG1) was probed against various SARS-CoV-2 RBDs. (c) Interaction between WT spike RBD monomer (blue column) and WT spike RBD trimer to CLEC2.Fc by ELISA. HC: healthy control. $^{**}p < 0.01$ (Student *t*-test).

Appendix Figure S2. Morphology of SARS-CoV-2-induced NET formation in the absence or presence of platelets. Human neutrophils (4×10^5/ml) were incubated with SARS-CoV-2 (MOI =1) in the absence (I) or presence (II) of platelets for 20 h at 37 $^{\circ}$C. Addition of anti-CLEC2 mAb inhibited SARS-CoV-2/platelet-induced NET formation (III). blue: DNA; red: MPO; green: Cit-H3; cyanogen: co-localization of DNA, MPO, and histone.

Appendix Figure S3. Detection of NETs on glass plates and MPO-Elastase-DNA complex in culture supernatant.

Human neutrophils were stimulated with PMA (100 nM) or SARS-CoV-2 spike RBD pseudotyped virus (pseudotyped virus) (MOI = 0.1) in presence of autologous platelets at 37˚C for 0.5 and 3 h. NETs on coverslip were observed using fluorescent staining of DNA (Blue), MPO (red), and citrullinated histone 3 (green), and the quantification of NETs on coverslip was using MPO/Cit-H3 colocalized area (mm^2) **(a)**. NET level in the supernatant was measured by MPO/elastase-DNA complex ELISA) **(b)**. *p <0.05, **p < 0.01 (Student's *t*-test).

Appendix Figure S4. Interaction between human CLEC2.Fc (hCLEC2.Fc) and mouse CLEC2.Fc (mCLEC2.Fc) to recombinant SARS-CoV-2 RBD.

Interaction between human CLEC2.Fc (hCLEC2.Fc), mouse CLEC2.Fc (mCLEC2.Fc) and recombinant SARS-CoV-2 RBD were determined by ELISA. **p < 0.01, ***p < 0.001 (Student *t*-test).

Appendix Figure S5. SARS-CoV-2 induces NET formation and thrombosis in heart from AAV-hACE2 mice. AAV-hACE2 mice were challenged with SARS-CoV-2 (8×10^4 PFU) and heart and spleen were harvested at day 5 post-infection. NET and immunothrombosis were visualized by stain with Hoechst33342 (blue), anti-Gr-1 antibody (red), anti-MPO antibody (green), anti-Cit-H3 antibody (cyanogen), and anti-CD42b antibody (yellow). (a&b) Heart from SARS-CoV-2-inoculated mice at day 5 post-infection was observed NET and immunothrombosis. Whole heart image scan was shown in **(a)** and higher magnification was shown in **(b)**. Scale bar 100 μ

m. (c) Spleen from SARS-CoV-2-inoculated mice at day 5 post-infection was not observed any NET or immunothrombosis. Scale bar 1 mm.

Appendix Figure S6. CLEC2.Fc treatment decreases the SARS-CoV-2-infection-induced NET and immunothrombosis in lung. AAV-hACE2 mice were challenged with SARS-CoV-2 (8×10^4 PFU). Lung was collected at day 5 post-infection, and tissue sections were stained with DAPI (blue), anti-MPO antibody (green), anti-citrullinated histone H3 antibody/Cit-H3 (red), anti-CD31 antibody (gray) and anti-CD42b antibody (yellow). Whole lung images were scaned by Leica confocal microscope with white light laser system (TCS SP8 X-FALCON). Scale bar is 1 mm.

Appendix Figure S7. Cell infiltration in SARS-CoV-2-infected mice. The infiltrated cell populations were stained with anti-CD11b antibody, anti-CD64 antibody, anti-F4/80 antibody, and anti-Ly6C antibody. Interstitial macrophages (interstitial MΦ) were defined as $CD11b^{+}CD64^{+}F4/80^{+}$; monocyte-derived dendritic cells (DC)/macrophages (MΦ) were defined as $CD11b^{+}CD64^{+}Ly6C^{+}$; $Ly6C^{+}$ monocytes were defined as $Ly6C^{+}$. The numbers of each cell population were calculated using the multiple fluorescent staining image analyzed by MetaMorph software. *$p < 0.05$, **$p < 0.01$, ****$p < 0.0001$ (Student's *t*-test).

Appendix Figure S8. The biodistribution of hIgG and CLEC2.Fc in C57BL/6 wild type mice. **(a-e)** 8 weeks old C57BL/6 mice were intravenously injected 1mCi of ^{111}In-DTPA-hCLEC2 Fc and 111In-hIgG1 (equivalent 10mg/kg of protein, n=3). Images **(a)** and blood samples **(b & c)** were collected at 0.5, 1, 2, 4, 24, 48, 96, and 168 h after injection. Mice were sacrificed by cervical vertebra dislocation at 168 h after administration. The NanoSPECT/CT (Mediso Medical Imaging Systems, USA) plus scanner system (a), the uptake of radioactivity was measured by a gamma

counter (PerkinElmer, Waltham, MA, USA) (b & c). Data are expressed as the percentage of injected dose per gram of organ (%ID/g) **(d)** or protein concentration per gram of organ (μg/g) **(e)**.

Appendix Figure S9. Expression of podoplanin in lung after SARS-CoV-2 infection. Lung from AAV/ACE-SARS-CoV-2-infected mice were collected at day 3 and day 5 post-inoculation. (a) The mRNA level of *pdpn* was measured by qPCR (normalized to uninfected mice/mock). (b) Tissue sections were stained with anti-podoplanin antibody, and the quantification of PDPN was calculated by MetaMorphTM software).

Appendix Figure S10. Both human podoplanin and murine podoplanin bind to human CLEC2. The interaction between CLEC2.Fc with SARS-CoV2 RBD, human podoplanin (PDPN), or mouse PDPN was measured by ELISA.

Appendix Figure S11. Construct of human CLEC2.Fc.

Restriction map of pcDNA3/human CLEC2-huIgG1.Fc. The mutated Fc fragment of human immunoglobulin G (hIgG) 1 (L234A, L235E, G237A, and P331S) was subcloned into pcDNA3 vector, and leaves multiple cloning sites in the N and C-termini, respectively. Primers for CLEC2 extracellular domain (ECD) cloning: forward: GGATCCCTGGGGATTTGGTCTGTC; reverse: GAATTCTTAAGGTAGTTGGTCCAC.

Appendix Table S1. Neutralizing assay of RBD neutralizing mAb and CLEC2.Fc

	Neutralization titer	
	$TCID_{10}$	$TCID_{100}$
**RBD neutralizing mAb**	1 nM	2 nM
**CLEC2.Fc**	> 1 mM	> 1 mM

For the microneutralization assay, serial 2-fold dilutions of RBD neutralizing mAb and CLEC2.Fc (starting at 2 mM) were mixed with equal volumes of 100 and 10 $TCID_{50}$ of SARS-CoV-2 (hCoV-19/Taiwan/4/2020 isolate). After 1 h of incubation at 37°C, 100 µl of the mixture was added in duplicate to confluent monolayers of Vero E6 cells in 96-well microtiter plates. The plates were then incubated for 3 more days at 37°C in 5% CO_2 humidified incubator. A virus back-titration was performed to assess input virus dose. Cytopathic effect (CPE) was observed at 3 days post infection. The highest dilution that completely protected the cells from CPE was recorded as the neutralization titer.

Appendix Table S2. Sequences of qPCR primers

Target	Forward sequence (5' to 3')	Reverse sequence (5' to 3')
mouse *tnf-α*	GCCTCTTCTCATTCCTGCTTG	CTGATGAGAGGGAGGCCATT
mouse *il-6*	GAGGATACCACTCCCAACAGACC	AAGTGCATCATCGTTGTTCATAC
mouse *il-1β*	GGAGAACCAAGCAACGACAAAATA	TGGGGAACTCTGCAGACTCAAAC
mouse *ip-10*	CCAAGTGCTGCCGTCATTTTC	GGCTCGCAGGGATGATTTCAA
mouse *cxcl1*	CAATGAGCTGCGCTGTCAGTG	CTTGGGGACACCTTTTAGCATC
mouse *cxcl2*	CCAAGGGTTGACTTCAAGAAC	AGCGAGGCACATCAGGTACG
mouse *cxcl5*	CCGCTGGCATTTCTGTTGCTGT	CAGGGATCACCTCCAAATTAGCG
mouse *ccl2*	TTAAAAACCTGGATCGGAACCAA	GCATTAGCTTCAGATTTACGGGT
mouse *ccl5*	GCTGCTTTGCCTACCTCTCC	TCGATGACAAACACGACTGC
mouse *ifn-γ*	CATGGCTGTTTCTGGCTGTTACTG	GTTGCTGATGGCCTGATTGTCTTT
mouse *il-10*	CTTGCACTACCAAAGCCACA	TAAGAGCAGGCAGCATAGCA
mouse *cramp*	GCCGCTGATTCTTTTGACAT	GCCAAGGCAGGCCTACTACT
mouse *gapdh*	GGAGAAACCTGCCAAGTATG	TGGGAGTTGCTGTTGAAG
