# Supplementary material for: Inhibition of SARS‐CoV‐2‐mediated thromboinflammation by CLEC2.Fc
Source: EMBO Mol Med. 2023 May 22;15(7):e16351. doi: 10.15252/emmm.202216351 (PMC10331576; doi:10.15252/emmm.202216351)
Supplement: Supplementary file 8 [file EMMM-15-e16351-s008.pdf]

EMBO Molecular Medicine

Inhibition of SARS-CoV-2-mediated thromboinflammation by CLEC2.Fc

Pei-Shan Sung, Cheng-Pu Sun, Mi-Hwa Tao, Shie-Liang Hsieh
DOI: 10.15252/emmm.202216351

Corresponding author: Shie-Liang Hsieh (slhsieh@gate.sinica.edu.tw)

Review Timeline:

Submission Date:	22nd May 22
Editorial Decision:	23rd Jun 22
Revision Received:	18th Apr 23
Editorial Decision:	3rd May 23
Revision Received:	5th May 23
Accepted:	5th May 23

Editor: Zeljko Durdevic

Transaction Report:

(Note: With the exception of the correction of typographical or spelling errors that could be a source of ambiguity, letters and reports are not edited. Depending on transfer agreements, referee reports obtained elsewhere may or may not be included in this compilation. Referee reports are anonymous unless the Referee chooses to sign their reports.)

23rd Jun 2022

Dear Dr. Hsieh,

Thank you for the submission of your manuscript to EMBO Molecular Medicine, and please accept my apologies for the delay in getting back to you. We have now received feedback from two of the three reviewers who agreed to evaluate your manuscript. Given that referee #2 will unfortunately not be able to return his/her report in a timely manner, and that both referees #1 and #3 gave similar recommendation, we prefer to make a decision now in order to avoid further delay in the process. Should referee #2 provide a report, we will send it to you, with the understanding that we will not ask for an additional revision. As you will see from the reports below, the referees acknowledge the interest of the study but also raise serious and partially overlapping concerns that should be addressed in a major revision.

Further consideration of a revision that addresses reviewers' concerns in full will entail a second round of review. EMBO Molecular Medicine encourages a single round of revision only and therefore, acceptance or rejection of the manuscript will depend on the completeness of your responses included in the next, final version of the manuscript. For this reason, and to save you from any frustrations in the end, I would strongly advise against returning an incomplete revision.

We would welcome the submission of a revised version within three months for further consideration. Please let us know if you require longer to complete the revision.

I look forward to receiving your revised manuscript.

Yours sincerely,

Zeljko Durdevic

Zeljko Durdevic
Editor
EMBO Molecular Medicine

*****

When submitting your revised manuscript, please carefully review the instructions that follow below. We perform an initial quality control of all revised manuscripts before re-review; failure to include requested items will delay the evaluation of your revision.

We require:

1) A .docx formatted version of the manuscript text (including legends for main figures, EV figures and tables). Please make sure that the changes are highlighted to be clearly visible.

2) Individual production quality figure files as .eps, .tif, .jpg (one file per figure). For guidance, download the 'Figure Guide PDF': (https://www.embopress.org/page/journal/17574684/authorguide#figureformat).

3) A .docx formatted letter INCLUDING the reviewers' reports and your detailed point-by-point responses to their comments. As part of the EMBO Press transparent editorial process, the point-by-point response is part of the Review Process File (RPF), which will be published alongside your paper.

4) A complete author checklist, which you can download from our author guidelines (https://www.embopress.org/page/journal/17574684/authorguide#submissionofrevisions). Please insert information in the checklist that is also reflected in the manuscript. The completed author checklist will also be part of the RPF.

5) Please note that all corresponding authors are required to supply an ORCID ID for their name upon submission of a revised manuscript.

6) It is mandatory to include a 'Data Availability' section after the Materials and Methods. Before submitting your revision, primary datasets produced in this study need to be deposited in an appropriate public database, and the accession numbers and database listed under 'Data Availability'. Please remember to provide a reviewer password if the datasets are not yet public (see https://www.embopress.org/page/journal/17574684/authorguide#dataavailability).

In case you have no data that requires deposition in a public database, please state so in this section. Note that the Data Availability Section is restricted to new primary data that are part of this study.

7) For data quantification: please specify the name of the statistical test used to generate error bars and P values, the number (n) of independent experiments (specify technical or biological replicates) underlying each data point and the test used to calculate p-values in each figure legend. The figure legends should contain a basic description of n, P and the test applied. Graphs must include a description of the bars and the error bars (s.d., s.e.m.). See also 'Figure Legend' guidelines: https://www.embopress.org/page/journal/17574684/authorguide#figureformat

8) We would also encourage you to include the source data for figure panels that show essential data. Numerical data should be provided as individual .xls or .csv files (including a tab describing the data). For blots or microscopy, uncropped images should be submitted (using a zip archive if multiple images need to be supplied for one panel). Additional information on source data and instruction on how to label the files are available at

.

9) Our journal encourages inclusion of *data citations in the reference list* to directly cite datasets that were re-used and obtained from public databases. Data citations in the article text are distinct from normal bibliographical citations and should directly link to the database records from which the data can be accessed. In the main text, data citations are formatted as follows: "Data ref: Smith et al, 2001" or "Data ref: NCBI Sequence Read Archive PRJNA342805, 2017". In the Reference list, data citations must be labeled with "[DATASET]". A data reference must provide the database name, accession number/identifiers and a resolvable link to the landing page from which the data can be accessed at the end of the reference. Further instructions are available at .

10) We replaced Supplementary Information with Expanded View (EV) Figures and Tables that are collapsible/expandable online. A maximum of 5 EV Figures can be typeset. EV Figures should be cited as 'Figure EV1, Figure EV2" etc... in the text and their respective legends should be included in the main text after the legends of regular figures.

- For the figures that you do NOT wish to display as Expanded View figures, they should be bundled together with their legends in a single PDF file called *Appendix*, which should start with a short Table of Content. Appendix figures should be referred to in the main text as: "Appendix Figure S1, Appendix Figure S2" etc.

- Additional Tables/Datasets should be labeled and referred to as Table EV1, Dataset EV1, etc. Legends have to be provided in a separate tab in case of .xls files. Alternatively, the legend can be supplied as a separate text file (README) and zipped together with the Table/Dataset file.

See detailed instructions here:

.

11) The paper explained: EMBO Molecular Medicine articles are accompanied by a summary of the articles to emphasize the major findings in the paper and their medical implications for the non-specialist reader. Please provide a draft summary of your article highlighting

- the medical issue you are addressing,

- the results obtained and

- their clinical impact.

This may be edited to ensure that readers understand the significance and context of the research. Please refer to any of our published articles for an example.

12) For more information: There is space at the end of each article to list relevant web links for further consultation by our readers. Could you identify some relevant ones and provide such information as well? Some examples are patient associations, relevant databases, OMIM/proteins/genes links, author's websites, etc...

13) Author contributions: the contribution of every author must be detailed in a separate section (before the acknowledgments).

14) A Conflict of Interest statement should be provided in the main text.

15) Every published paper now includes a 'Synopsis' to further enhance discoverability. Synopses are displayed on the journal webpage and are freely accessible to all readers. They include a short stand first (maximum of 300 characters, including space) as well as 2-5 one-sentences bullet points that summarizes the paper. Please write the bullet points to summarize the key NEW findings. They should be designed to be complementary to the abstract - i.e. not repeat the same text. We encourage inclusion of key acronyms and quantitative information (maximum of 30 words / bullet point). Please use the passive voice. Please attach these in a separate file or send them by email, we will incorporate them accordingly.

Please also suggest a striking image or visual abstract to illustrate your article as a PNG file 550 px wide x 300-600 px high.

EMBO Molecular Medicine has a "scooping protection" policy, whereby similar findings that are published by others during review or revision are not a criterion for rejection. Should you decide to submit a revised version, I do ask that you get in touch after three months if you have not completed it, to update us on the status.

***** Reviewer's comments *****

Referee #1 (Comments on Novelty/Model System for Author):

The study is well done using state of the art methods but the clinical significance is negligible due to the low affinity of rec CLEC-2 Fc and therefore off-target effects, unclear significance of blocking thrombo-inflammation in these patients, and the availability of alternative treatments. It would be hard to see the justification for a clinical trial

Referee #1 (Remarks for Author):

In this study, the authors show that CLEC-2 binds to the receptor binding domain in SARS-COV-2 spike protein and that this interaction on platelets can enhance formation of NETS by neutrophils in vitro and in vivo. This does not occur in CLEC-2 deficient mice and is inhibited by recombinant CLEC-2 in vivo. The authors report the affinity of binding of SARS-COV-2 to Fc-CLEC-2 is in the order of 10 M which is a relatively low value and so off-target effects are a concern. The authors propose that rCLEC-2-Fc could be used to prevent immunothrombosis in patients with Covid-19. The authors show that rCLEC-2-Fc does not alter virus infection.

Overall

The research is performed to a high level and the observations are convincing, but the clinical significance would appear to be minimal due to the low affinity of the interaction of rCLEC-2 and the spike protein, and availability of alternative ways to prevent thrombo-inflammatory events: further, the effectiveness of preventing thrombo-inflammatory events in patients with Covid-19 is unclear.

Major

1. The reliant on histone staining for next quantitation (e.g. (fig 3) should be extended to another marker e.g. MPO/ DNA staining, especially as this is not clearly visible in fig 5. There is variability in the NET histone area quantitation in figure 3a and b, noting the difference in axis. The isotype in part 3b should be near the 8000 mark like in part 3a, but it is 2000.

Minor

1. The comparison to the affinity to podoplanin does not take into account that podoplanin is expressed on a surface and therefore it mediates its effects through affinity and avidity. How many copies of spike protein are present on the virus?
2. The use of 'home-made' to describe expression of CLEC-2-Fc in an article of this nature is out of place.
3. Reference required for CLEC-2 expression levels rather than say expressed abundantly (p4 line66/67)
4. Clarify what is meant by 'microclots' in the intro. Clot with fibrin and wbc or platelet rich microaggregates etc.
5. Missing l in black page p6 line 131.

Referee #3 (Comments on Novelty/Model System for Author):

In this work, the authors investigated the underlying molecular cause of the severe acute respiratory syndrome coronavirus 2 (SARS-CoV-2)-mediated thromboinflammation and identified the platelet C-type lectin member 2 (CLEC2) receptor as a putative binding partner for SARS-CoV-2 spike protein receptor-binding domain (RBD). To this end, the authors first demonstrated the capacity of CLEC2 to interact with the SARS-CoV-2 RBD. Enzyme linked immunosorbent assay (ELISA)-based assays, as well as bio-layer-interferometry showed, that CLEC2/ CLEC2.Fc binds to the SARS-CoV-2 RBD with high affinity. In vitro Neutrophil extracellular trap (NET) formation in human neutrophils 5h and 20h post-infection with SARS-CoV-2 was measured using microscopic approaches with immunofluorescent staining, and SARS-CoV-2 alone was able to induce robust NET formation. Coincubation with autologous platelets lead to substantially increased NET formation in this approach, but at 20h post-infection, the NETs detached, putatively leading to an artificially lower signal. Moreover, anti-CLEC2 mAb was able to revert these effects, showing that platelet CLEC2 was the underlying cause for the enhanced NET formation. Murine neutrophils were then challenged with SARS-CoV-2 for NET formation in the presence of wildtype or clec2-/- platelets, and, contrary to wildtype platelets, almost no NET formation was detectable using clec2-/- platelets. Infection with a SARS-CoV-2 spike pseudotyped lentivirus lead to enhanced NET formation in human neutrophils in the presence of platelets, and here again, anti-CLEC2 mAb was able to abolish these effects, showing that SARS-CoV-2 spike RBD binding to CLEC2 is responsible for this. CLEC2.Fc inhibited NET formation in human neutrophils upon incubation with SARS-CoV-2 in vitro, as well as platelet-mediated enhanced NET formation. Infecting AAV-ACE2-mice with SARS-CoV-2 and administration of CLEC2.Fc significantly lowered the expression of proinflammatory cytokines and chemokines in a prophylactic, as well as in a therapeutic model, whilst not being able to reduce virus titers, as assessed with real-time qPCR. CLEC2.Fc was also able to reduce NET formation (MPO+ area), as well as thrombus formation (CD42b+ area) and infiltration of other immune cells in murine lungs in comparison to saline-treated and isotype-treated animals, whereas the prophylactic model seems to be more promising than therapeutic model. Collagen deposition in the murine lung was significantly lower than isotype-treated animals' lungs after administration of CLEC2.Fc (prophylactic and therapeutic).
The manuscript is generally well written and structured, yet some questions arise while reading it. Still, the results may be of potential interest to the scientific community, as CLEC2.Fc may be a new therapeutic option regarding SARS-CoV-2 infection and long-COVID syndrome.

Referee #3 (Remarks for Author):

These are my specific issues:

Major points:

• What exactly is CLEC2.Fc and what is it supposed to do (why is it linked to an Fc region)? This is quite an important protein, used in various of the experiments and it also provides the option for therapeutic use, yet an explanation is not provided in the entire introduction/results part. This message is not delivered clearly enough and it may not be self explanatory to some readers, so could the authors please include a short introduction?
• Line 134: The authors of the manuscript describe the phenotype of the NETs they observed as aggregated NETs and report about detached NETs from the coverslips, but fail to show the detached NETs. The supplementary figure 2 shows the status quo at 20h post infection, and here it just seems that the virus alone induces robust aggregated NETs and the platelets + virus induce thread-like NETs. Why would the NETs detach, what makes them unstable? Is the NET composition different, when platelets are included in the experiment? Any speculation here? What is the status quo at 5h post-infection, for comparison (please include the pictures)? Why are these pictures in the supplement and not conjointly shown with the graphs in figure 3? For the reader's understanding it would be better to show them together with the respective quantification. A DNA-complex ELISA for NET detection using supernatant with the "floating NETs" from this experiment could provide further insight into this matter. If the NETs detach into the supernatant, the signal should be higher than with the virus alone.
• It is not clear, how exactly NET formation in vitro was quantified. What did the authors determine as NETs? This information is needed to understand what "the histone area of NETs" (line 358) is. How many sections/ fields of view were used for quantification? Also: why did the authors choose to examine for NETs at 5h and 20h post-incubation? It is unlikely, that a neutrophil survives for 20h in culture conditions, so the neutrophils may have just undergone some other cell death forms (apoptosis/ necrosis) at this point?
• Line 194 and Figure 5a,b: How were the NETs in vivo determined? Did the authors measure NET formation in the lung tissue only as MPO+ area (line 194)? State of the art in immunostainings of NETs is a colocalization of (extracellular) DNA with a typical NET protein (such as MPO or neutrophil elastase) and especially in infectious backgrounds, colocalization of a NET protein with citrullinated histone H3. Why was this not done/ shown? Figure 5a: Instead of using Hoechst 33342 to stain DNA, the authors could have used an endothelial cell marker and a neutrophil-specific marker (for instance Ly6G) to show neutrophil infiltration into the tissue. Why was this not done? MPO alone may have been released by neutrophils as one of their effector functions, without necessarily being a component of NETs, or it may even only reflect a massive neutrophil infiltration. It is very difficult to conclude if MPO+ area really reflects NET formation based on the provided data.
• Figure 5d: Why did the authors choose to skip the saline-treated control? It was done for assessment of NET formation (5b,c), so why not here? And why does it say then "saline" instead of "isotype" in Figure 5e? Please explain and harmonise.
• How was the animals' wellbeing, especially the virus-challenged saline-treated mice vs. the CLEC2.Fc-treated ones? Weight-loss? Survival-curves? Especially if CLEC2.Fc were to be used as a prophylactic/ therapeutic, this is valuable information and it would be good to provide this in the manuscript as well.

• Does the application of CLEC2.Fc affect any other tissue/ cell types and interfere with important molecular mechanisms? What is the half-life of this protein in mice, once administered? This should be addressed experimentally and also included in the discussion.

Minor points:

• Please explain the genetic background of the AAV-ACE2-mice and what is special about these mice, or rather why you chose to use this strain. What does the abbreviation AAV mean? Are those the same mice as the AAV-hACE2-mice?
• Line 99 ff: Please elaborate more on the CLEC2 that was used in these experiments. Human/ murine? Recombinant? In line 104, the authors now write about CLEC2.Fc, is that the same protein as CLEC2? Also, "homemade" (SARS-CoV-2 RBD) is not a valid scientific expression. Please be a little bit more specific.
• Line 100/ supplementary Fig. 1a: there is no red column in the supplementary figure 1a.
• Line 191 ff: The authors fail to mention where NET formation and thrombus formation was assessed in vivo. Was it done in the lung or did the authors also measure thrombus formation in other tissues?
• Figure 5a: Could the authors show sections of the microscopic pictures in higher magnification? There is almost no CitH3 signal (or it is at least not really visible in this magnification). Also, how many sections/ fields of view were used for quantification of NETs (MPO+ area)?
• Figure 5c: "mock" and "saline" dots have the same color, thus can't be differentiated.
• Line 193 ff and Figure 5b: "The quantitation of NET formation... was shown in Fig. 5b and 5c.". Phrases like these do not provide any information. Could the authors maybe describe the results and add the figure as a reference in brackets, as is usually done? Also: What is isotype? Does it refer to an antibody isotype control? Is it the same as mentioned in figure 4 description?
• Entire methods section: Please give a brief description of the method (it does not have to be long), even when using "as described (previously)" and referring to another paper. It is a nuisance to the reader when everything has to be looked up in other manuscripts. Just imagine you would like to understand a method but every paper omits the necessary information by writing " as described". In the worst case, you will go down a rabbit hole, without ever finding the information you were looking for.
• Line 355 ff/ Fig. 3 and Fig. 4a,b: Please name the exact working concentration instead of the dilution of the antibodies and reagents mentioned (anti-MPO, anti-citrullinated histone, Hoechst 33342).
• Line 364: Which primary (and secondary) antibodies were used here?
• Line 526 ff: Please harmonise the position of the sub-category letters ((a), (b),..) introducing the graph descriptions. This way, it's confusing and the descriptions do not always match the graphs. For instance, (a) is not the graph representing the platelets pretreated with anti-CLEC2 mAb.
• Supplementary figure 1b: Blue point is described with SARS, which would be the disease. Do the authors mean SARS-CoV?
• Supplementary figure 2: There is no color coding for the staining written underneath or above the pictures. It would be easier for the reader's understanding if included in the pictures than in the figure description.
• Reference 39 is just submitted and yet unpublished, which means that there is currently no way to retrace or comprehend the data referred to in lines 168 and 333.

Referee #1 (Comments on Novelty/Model System for Author):

The study is well done using state of the art methods but the clinical significance is negligible due to the low affinity of rec CLEC-2 Fc and therefore off-target effects, unclear significance of blocking thrombo-inflammation in these patients, and the availability of alternative treatments. It would be hard to see the justification for a clinical trial

Referee #1 (Remarks for Author):

In this study, the authors show that CLEC-2 binds to the receptor binding domain in SARS-COV-2 spike protein and that this interaction on platelets can enhance formation of NETS by neutrophils in vitro and in vivo. This does not occur in CLEC-2 deficient mice and is inhibited by recombinant CLEC-2 in vivo. The authors report the affinity of binding of SARS-COV-2 to Fc-CLEC-2 is in the order of 10 uM which is a relatively low value and so off-target effects are a concern. The authors propose that rCLEC-2-Fc could be used to prevent immunothrombosis in patients with Covid-19. The authors show that rCLEC-2-Fc does not alter virus infection.

Overall

The research is performed to a high level and the observations are convincing, but the clinical significance would appear to be minimal due to the low affinity of the interaction of rCLEC-2 and the spike protein, and availability of alternative ways to prevent thrombo-inflammatory events: further, the effectiveness of preventing thrombo-inflammatory events in patients with Covid-19 is unclear.

Major

1. The reliant on histone staining for next quantitation (e.g. (fig 3) should be extended to another marker e.g. MPO/ DNA staining, especially as this is not clearly visible in fig 5. There is variability in the NET histone area quantitation in figure 3a and b, noting the difference in axis. The isotype in part 3b should be near the 8000 mark like in part 3a, but it is 2000.

Ans:

- We follow the instruction to detect DNA/MPO/citruillinated histone to define the NET formation and show the image in Fig. 3a, and the quantitation change to citrullinated histone in Fig. 3b~e (Figure 3).

- The variation of SARS-CoV-2-induced NET formation is due to the differential responses of differnet individuals. It is common that the same batch of viruses with same MOI elicit differential responses of immune cells isolated from differnet individuals. The responses of human samples are more hetergenous than that using samples from inbred mouse strains.

Minor

1. The comparison to the affinity to podoplanin does not take into account that podoplanin is expressed on a surface and therefore it mediates its effects through affinity and avidity. How many copies of spike protein are present on the virus?

Ans:

According to previous studies, one SARS-CoV-2 contains 25-50 spike proteins (https://doi.org/10.1016/j.cell.2020.09.018), and each infected person carries 10^9 to 10^{11} virions during peak infection, with a total mass in the range of 1 μg to 100 μg (https://doi.org/10.1073/pnas.202481511).

2. The use of 'home-made' to describe expression of CLEC-2-Fc in an article of this nature is out of place.

Ans:

1) The production of CLEC2.Fc fusion protein was as described in the Appendix Table S1 (it was named as CLEC1B, https://www.nature.com/articles/nature07013#Sec21) of our previous publication (doi:10.1038/nature07013).

2) We added the description and map of CLEC2.Fc in the materials and methods and Appendix Figure S11.

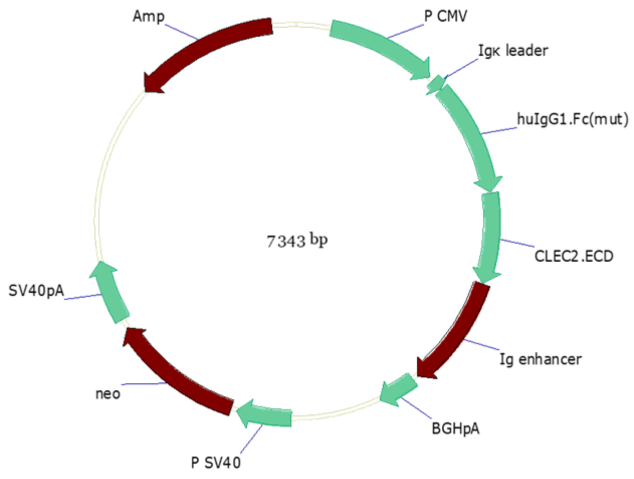

pcDNA3/huIgG1.Fc(mut)-huCLEC2.ECD

3. Reference required for CLEC-2 expression levels rather than say expressed abundantly (p4 line66/67)

Ans:

We add the reference as shown in line 82.

4. Clarify what is meant by 'microclots' in the intro. Clot with fibrin and wbc or platelet rich microaggregates etc.

Ans:

The term 'microclot' is quoted from reference (Pretorius et al, 2021, line 57). It is described as 'pellet deposits' comprising aggregated proteins resistant totrypsin digestion in the plasma. We added the descripton in the Introduction (line 56).

5. Missing l in black page p6 line 131.

Ans:

We correct the typo (line 161, b**l**ack column)

Referee #3 (Comments on Novelty/Model System for Author):
In this work, the authors investigated the underlying molecular cause of the severe acute respiratory syndrome coronavirus 2 (SARS-CoV-2)-mediated thromboinflammation and identified the platelet C-type lectin member 2 (CLEC2) receptor as a putative binding partner for SARS-CoV-2 spike protein receptor-binding domain (RBD). To this end, the authors first demonstrated the capacity of CLEC2 to interact with the SARS-CoV-2 RBD. Enzyme linked immunosorbent assay (ELISA)-based assays, as well as bio-layer-interferometry showed, that CLEC2/ CLEC2.Fc binds to the SARS-CoV-2 RBD with high affinity. In vitro Neutrophil extracellular trap (NET) formation in human neutrophils 5h and 20h post-infection with SARS-CoV-2 was measured using microscopic approaches with immunofluorescent staining, and SARS-CoV-2 alone was able to induce robust NET formation. Coincubation with autologous platelets lead to substantially increased NET formation in this approach, but at 20h post-infection, the NETs detached, putatively leading to an artificially lower signal. Moreover, anti-CLEC2 mAb was able to revert these effects, showing that platelet CLEC2 was the underlying cause for the enhanced NET formation. Murine neutrophils were then challenged with SARS-CoV-2 for NET formation in the presence of wildtype or clec2-/- platelets, and, contrary to wildtype platelets, almost no NET formation was detectable using clec2-/- platelets. Infection with a SARS-CoV-2 spike pseudotyped lentivirus lead to enhanced NET formation in human neutrophils in the presence of platelets, and here again, anti-CLEC2 mAb was able to abolish these effects, showing that SARS-CoV-2 spike RBD binding to CLEC2 is responsible for this. CLEC2.Fc inhibited NET formation in human neutrophils upon incubation with SARS-CoV-2 in vitro, as well as platelet-mediated enhanced NET formation. Infecting AAV-ACE2-mice with SARS-CoV-2 and administration of CLEC2.Fc significantly lowered the expression of proinflammatory cytokines and chemokines in a prophylactic, as well as in a therapeutic model, whilst not being able to reduce virus titers, as assessed with real-time qPCR. CLEC2.Fc was also able to reduce NET formation (MPO+ area), as well as thrombus formation (CD42b+ area) and infiltration of other immune cells in murine lungs in comparison to saline-treated and isotype-treated animals, whereas the prophylactic model seems to be more promising than therapeutic model. Collagen deposition in the murine lung was significantly lower than isotype-treated animals' lungs after administration of CLEC2.Fc (prophylactic and therapeutic).
The manuscript is generally well written and structured, yet some questions arise while reading it. Still, the results may be of potential interest to the scientific community, as CLEC2.Fc may be a new therapeutic option regarding SARS-CoV-2 infection and long-COVID syndrome.

Referee #3 (Remarks for Author):
These are my specific issues:

Major points:

• What exactly is CLEC2.Fc and what is it supposed to do (why is it linked to an Fc region)? This is quite an important protein, used in various of the experiments and it also provides the option for therapeutic use, yet an explanation is not provided in the entire introduction/results part. This message is not delivered clearly enough and it may not be self explanatory to some readers, so could the authors please include a short introduction?

Ans:

1) The production of CLEC2.Fc fusion protein was as described in the materials and methods (line 392-396), and the map was shown in Appendix Figure S11 (it was named as CLEC1B, https://www.nature.com/articles/nature07013#Sec21) of our previous publication (doi:10.1038/nature07013).

2) CLEC2.Fc has been shown to prevent accumulation and retention of inflammatory macrophages in LPS-induced pertonitis (doi: 10.3389/fimmu.2021.693974). We add several sentences to introduce the functions of CLEC2.Fc to inhibit inflammatory reaction in the INTRODUCTION (line 85-89). We add more sentences to discuss the potential mechanism of CLEC2.Fc-mediated effect in the SARS-CoV-2-induced thromoinflammation in the DISCUSSION (line 302-311).

• Line 134: The authors of the manuscript describe the phenotype of the NETs they observed as aggregated NETs and report about detached NETs from the coverslips, but fail to show the detached NETs. The Appendix figure 2 shows the status quo at 20h post infection, and here it just seems that the virus alone induces robust aggregated NETs and the platelets + virus induce thread-like NETs. Why would the NETs detach, what makes them unstable?

Ans:

In Fgigure 3a & 3b, we showed that platelet enhance virus-induced aggregated NET formation at 5 hr post incubation. At 20 h post-incubation, only few NETs are observed on the glass platelets (virus + platelets). We detected the DNA-MPO-elastase complex in supernatant by ELISA (Appendix Figure S3c), and find the amount of DNA-MPO-elastase complex induced by virus+platelets are much higher than virus alone, indicating more NET structures are release to culture supernatant.

It is still unknown why NETs detach from glass plate. However, our observatioin is in accord with the reports that the levels of cell-free DNA, MPO-DNA complex, and citrullinated histone H3 (Cit-H3) are elevated in blood samples of COVID-19 patients (Zuo et al, 2020, line766). We point out this issue in the DISCUSSION (line 291-295). It needs more works to answer this question in the future.

Is the NET composition different, when platelets are included in the experiment? Any speculation here?

Ans:

The classical defintion of NETs structures are composed on DNA, MPO, and citrulinated Histone from over-activated neutrophils. However, neutrophils and platelets adhere to each other after incubation with SARS-CoV-2, so the NETs stucture contains the componetns from both activated neutrophils and platelets, and is differnet from the classical NET strucuture.

What is the status quo at 5h post-infection, for comparison (please include the pictures)?

Ans:

We include images of 5 h and 20 h post-infection in this revised version (Fig.3a).

Why are these pictures in the supplement and not conjointly shown with the graphs in figure 3?

Ans:

We follow your instruction and move the pictures to Fig, 3a in this revised version, and the original quantitation data is changed to Fig. 3b in this revised version.

For the reader's understanding it would be better to show them together with the respective quantification. A DNA-complex ELISA for NET detection using supernatant with the "floating NETs" from this experiment could provide further insight into this matter. If the NETs detach into the supernatant, the signal should be higher than with the virus alone.

Ans:

(a) We follow the instruction to detect the DNA-MPO-elastase complex in supernatant by ELISA (Appendix figure S3c).
(b) Indeed, the DNA-MPO-elastase complex is much higher in virus+platelets than virus alone (Appendix figure S3c).

• It is not clear, how exactly NET formation in vitro was quantified. What did the authors determine as NETs? This information is needed to understand what "the histone area of NETs" (line 358) is. How many sections/ fields of view were used for quantification? Also: why did the authors choose to examine for NETs at 5h and 20h post-incubation? It is unlikely, that a neutrophil survives for 20h in culture conditions, so the neutrophils may have just undergone some other cell death forms (apoptosis/ necrosis) at this point?

Ans:

1) In this revised version, we used the citrulinated histone to define the area of NET formation in all the figures, and detected other markers (DNA, MPO and others) to define NET area.

2) We analyze at least five fields of each sample and repeat 3 times from 3 independent experiments.

3) According to a previous study, HIV induces NET formation at 24 h post-incubation (https://doi.org/10.1016/j.chom.2012.05.015), even though most viruses induce NET formation in a short incubation period (1 to 3 hr) (table I, https://doi.org/10.1186/s12929-021-00741-7).

4) We have no idea how long the SARS-CoV-2 can induce NET formation in the beginning of this study, so we decide to observe in a shorter incubation time (5 h) and a longer incubation time (20 h) to check the level of NET formation.

5) Previous studies indicated that upon inflammation, various factors (pro-inflammatory cytokines, DAMPs, PAMPs, growth factors, etc) will stimulate neutrophils and prolong their lifespan (https://doi.org/10.1134/S0022093021030017).

• Line 194 and Figure 5a,b: How were the NETs in vivo determined? Did the authors measure NET formation in the lung tissue only as MPO+ area (line 194)?
State of the art in immunostainings of NETs is a colocalization of (extracellular) DNA with a typical NET protein (such as MPO or neutrophil elastase) and especially in infectious backgrounds, colocalization of a NET protein with citrullinated histone H3. Why was this not done/ shown?

Ans:

we use the whole lung image to measure the MPO^{+}/Cit-$H3^{+}$ colocalized area (as NET formation), and $CD42b^{+}$/Cit-$H3^{+}$ colocalized area as immunothrombosis in figure 5. We also detect the presence of immunothromobosis in heart (Appendix Fig. S5)

Figure 5a: Instead of using Hoechst 33342 to stain DNA, the authors could have used an endothelial cell marker and a neutrophil-specific marker (for instance Ly6G) to

show neutrophil infiltration into the tissue. Why was this not done? MPO alone may have been released by neutrophils as one of their effector functions, without necessarily being a component of NETs, or it may even only reflect a massive neutrophil infiltration. It is very difficult to conclude if MPO+ area really reflects NET formation based on the provided data.

Ans:

We include the endothelial cell marker **(CD31) and other markers (Gr1, DNA, Cit-H3, MPO, CD42b**) to detect NETs ($DNA^+Gr\text{-}1^+MPO^+Cit\text{-}H3^+$) and immunothrombosis (NETs + thrombus($CD42b^+$) in lung (Fig. 5a and legend) in this revised version.

• Figure 5d: Why did the authors choose to skip the saline-treated control? It was done for assessment of NET formation (5b,c), so why not here? And why does it say then "saline" instead of "isotype" in Figure 5e? Please explain and harmonise.

Ans:

We include the **saline-treated control and the isotype image** in this revised version (Figre 6b & 6c).

• How was the animals' wellbeing, especially the virus-challenged saline-treated mice vs. the CLEC2.Fc-treated ones? Weight-loss? Survival-curves? Especially if CLEC2.Fc were to be used as a prophylactic/ therapeutic, this is valuable information and it would be good to provide this in the manuscript as well.

Ans:

1) The descriptioin of the animal model and releated information is as described in the paper published by Sun et al., 2021(line 709).

2) All the mice surviv and would not die after infection. There is no obvious weight loss in this mouse model system (as shown in the following figure).

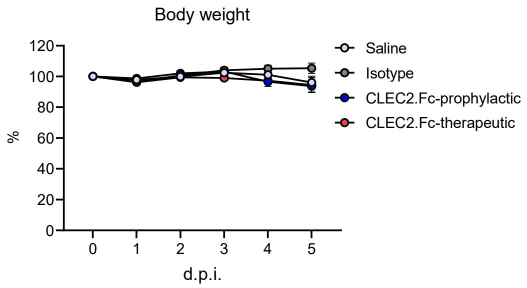

• Does the application of CLEC2.Fc affect any other tissue/ cell types and interfere with important molecular mechanisms? What is the half-life of this protein in mice,

once administered? This should be addressed experimentally and **also included in the discussion.**

Ans:

We follow the instruction and perform the experiment to understand the pharmacokinetics (PK) and biodistribution of CLEC2.Fc in vivo (line 250-259, Appendix figure 8). The half-life for hIgG1 and CLEC2.Fc is 61.04 h (±7.45) and 35.15 h (±2.89), respectively (calculated from Appendix figure 8c).

Minor points:

• Please explain the genetic background of the AAV-ACE2-mice and what is special about these mice, or rather why you chose to use this strain. What does the abbreviation AAV mean? Are those the same mice as the AAV-hACE2-mice?

Ans:

1) Yes, the AAV means the AAV-hACE2 mice. The descriptioin of the animal model and releated information is as described in the in the paper published by Sun et al., 2021(line 709).

2) The reason we choose this model is becuase mouse model mimics the pathophysiology of COVID-19 patients (line 201-203, Sun et al., 2021, line 709)

• Line 99 ff: Please elaborate more on the CLEC2 that was used in these experiments. Human/ murine? Recombinant? In line 104, the authors now write about CLEC2.Fc, is that the same protein as CLEC2? Also, "homemade" (SARS-CoV-2 RBD) is not a valid scientific expression. Please be a little bit more specific.

Ans:

We also add a sentence to describe CLEC2.Fc in this revised version (line 82-86). Except from the Taiwan strain, all the other RBDs of SARS-CoV-2 WT and variants are pruchased from the Acro Biosystem (line 369-372).

• Line 100/ Appendix Fig. 1a: there is no red column in the Appendix figure 1a.

Ans:

The Appendix Fig. 1a becomes Fig. 1a. We correct the typo and the description is in line 124 of this revised version (Fig. 1a).

• Line 191 ff: The authors fail to mention where NET formation and thrombus formation was assessed in vivo. Was it done in the lung or did the authors also measure thrombus formation in other tissues?

Ans:
We observed NET and immunothrombosis (NET+thrombus) in lung (Fig. 5) and heart (Appendix Figure S5a & S5b) from SARS-CoV-2-infected mice at day 5 post-infection. However, SARS-CoV-2 infection does not increase the NET and thrombus in spleen (Appendix Figure S5c).

• Figure 5a: Could the authors show sections of the microscopic pictures in higher magnification? There is almost no CitH3 signal (or it is at least not really visible in this magnification). Also, how many sections/ fields of view were used for the quantification of NETs (MPO+ area)?
Ans:
We show the high magnification of sections in the updated Fig.5a. We use the whole lung image to measure the MPO^+/Cit-$H3^+$ colocalized area asNET formation level (Fig.5b), and $CD42b^+$/Cit-$H3^+$ colocalized area as immunothrombosis (Fig.5c).

• Figure 5c: "mock" and "saline" dots have the same color, thus can't be differentiated.
Ans:
We change the color of Mock (white) and saline (light blue) in this revised version (Fig. 6b).

• Line 193 ff and Figure 5b: "The quantitation of NET formation... was shown in Fig. 5b and 5c.". Phrases like these do not provide any information. Could the authors maybe describe the results and add the figure as a reference in brackets, as is usually done?
Ans:
The description of figure 5b and 5c is rephrased in this revised versioin (line 224-237).

Also: What is isotype? Does it refer to an antibody isotype control? Is it the same as mentioned in figure 4 description?
Ans:
We change 'isotype' into 'hIgG1' in the Figure 4 and legend in this revised version.

• Entire methods section: Please give a brief description of the method (it does not have to be long), even when using "as described (previously)" and referring to another paper. It is a nuisance to the reader when everything has to be looked up in other manuscripts. Just imagine you would like to understand a method but every paper

omits the necessary information by writing " as described". In the worst case, you will go down a rabbit hole, without ever finding the information you were looking for.

Ans:

we add several sentences to describe the method briefly in this revised version (line 393-396; line 414-421; line 429-437; line 441-443).

• Line 355 ff/ Fig. 3 and Fig. 4a,b: Please name the exact working concentration instead of the dilution of the antibodies and reagents mentioned (anti-MPO, anti-citrullinated histone, Hoechst 33342).

Ans:

We replace the 'dilution' with exact concnentration (highlighted in blue color) of antibodies and DNA in this revised version (line 467-468).

• Line 364: Which primary (and secondary) antibodies were used here?

Ans:

We add the information of primar and secondary antibodies in the Materials and methods section- "Reagents and antibodies" in this revised version (Line 351-366).

• Line 526 ff: Please harmonise the position of the sub-category letters ((a), (b),..) introducing the graph descriptions. This way, it's confusing and the descriptions do not always match the graphs. For instance, (a) is not the graph representing the platelets pretreated with anti-CLEC2 mAb.

Ans:

We follow the instruction and rephrase the legend for figure 3 in this revised version (line 790-809).

• Appendix figure 1b: Blue point is described with SARS, which would be the disease. Do the authors mean SARS-CoV?

Ans:

We change the lable SARS into SARS-CoV to prevent confusion (it becomes Appendix Fig. S1a in this revsied figure). We compared the binding of CLEC2.Fc to the RBD from SARS-CoV and SARS-CoV-2.

• Appendix figure 2: There is no color coding for the staining written underneath or above the pictures. It would be easier for the reader's understanding if included in the pictures than in the figure description.

Ans:

We add arrow heads to indicate the DNA, MPO, and cit-histone on the figure (it is changed into Fig. 3a in this revised version.

• Reference 39 is just submitted and yet unpublished, which means that there is currently no way to retrace or comprehend the data referred to in lines 168 and 333.

Ans:

It has been published in the PLOS Pathogens (PLoS Pathog 17: e1009758, line 709)

3rd May 2023

Dear Dr. Hsieh,

Thank you for the submission of your revised manuscript to EMBO Molecular Medicine. I am pleased to inform you that we will be able to accept your manuscript pending the following final amendments:

1) Please address the referee #1 concern regarding low medical impact by discussing limited clinical implications of the study at this stage and future directions towards potential clinical translation.
2) In the main manuscript file, please do the following:
- In M&M, please specify the biosafety level for the experiments with SARS-CoV-2 by adding and amending the following sentence: All experiments with SARS-CoV-2 were performed in a ... level laboratory and with approval from...
- In M&M, provide the antibody dilutions that were used for each antibody.
- In M&M, please include statement that the informed consent was obtained from all human subjects and that the experiments conformed to the principles set out in the WMA Declaration of Helsinki and the Department of Health and Human Services Belmont Report.
- In M&M, add statistical paragraph that should reflect all information that you have filled in the Authors Checklist, especially regarding randomization, blinding, replication.
- Data availability statement should contain information about data, produced in this study, that cannot be published in the manuscript itself (e.g. structural data, high-throughput sequencing or data from large-scale gene expression experiments). If no data, produced in this study, are deposited in public repositories, please add the sentence: "This study includes no data deposited in external repositories" and remove the current sentences.
3) The Paper Explained: Please add it to the main manuscript file and structure it as followed: PROBLEM - the medical issue you are addressing, RESULTS - the results obtained, and IMPACT - their clinical impact. Please refer to any of our published primary research articles for an example. Please check "Author Guidelines" for more information.
https://www.embopress.org/page/journal/17574684/authorguide#researcharticleguide
4) Synopsis: Please check your synopsis text and image before submission with your revised manuscript. Please be aware that in the proof stage minor corrections only are allowed (e.g., typos).
5) For more information: This space should be used to list relevant web links for further consultation by our readers. Could you identify some relevant ones and provide such information as well? Some examples are patient associations, relevant databases, OMIM/proteins/genes links, author's websites, etc...
6) Source data: We encourage you to include the source data for figure panels that show essential data. Numerical data should be provided as individual .xls or .csv files (including a tab describing the data). For blots or microscopy, uncropped images should be submitted (using a zip archive if multiple images need to be supplied for one panel). Please check "Author Guidelines" for more information. https://www.embopress.org/page/journal/17574684/authorguide#sourcedata
7) As part of the EMBO Publications transparent editorial process initiative (see our Editorial at http://embomolmed.embopress.org/content/2/9/329), EMBO Molecular Medicine will publish online a Review Process File (RPF) to accompany accepted manuscripts. This file will be published in conjunction with your paper and will include the anonymous referee reports, your point-by-point response and all pertinent correspondence relating to the manuscript. Let us know whether you agree with the publication of the RPF and as here, if you want to remove or not any figures from it prior to publication. Please note that the Authors checklist will be published at the end of the RPF.
8) Please provide a point-by-point letter INCLUDING my comments as well as the reviewer's reports and your detailed responses (as Word file).

I look forward to reading a new revised version of your manuscript as soon as possible.

Yours sincerely,

Zeljko Durdevic

Zeljko Durdevic
Editor
EMBO Molecular Medicine

*** Instructions to submit your revised manuscript ***

*** PLEASE NOTE *** As part of the EMBO Publications transparent editorial process initiative (see our Editorial at

https://www.embopress.org/doi/pdf/10.1002/emmm.201000094), EMBO Molecular Medicine will publish online a Review Process File to accompany accepted manuscripts.

In the event of acceptance, this file will be published in conjunction with your paper and will include the anonymous referee reports, your point-by-point response and all pertinent correspondence relating to the manuscript. If you do NOT want this file to be published, please inform the editorial office at contact@embomolmed.org.

When submitting your revised manuscript, please include:

1) a .docx formatted version of the manuscript text (including Figure legends and tables)

2) Separate figure files*

3) supplemental information as Expanded View and/or Appendix. Please carefully check the authors guidelines for formatting Expanded view and Appendix figures and tables at
https://www.embopress.org/page/journal/17574684/authorguide#expandedview

4) a letter INCLUDING the reviewer's reports and your detailed responses to their comments (as Word file).

5) The paper explained: EMBO Molecular Medicine articles are accompanied by a summary of the articles to emphasize the major findings in the paper and their medical implications for the non-specialist reader. Please provide a draft summary of your article highlighting
- the medical issue you are addressing,
- the results obtained and
- their clinical impact.
This may be edited to ensure that readers understand the significance and context of the research.
Please refer to any of our published articles for an example.

6) For more information: There is space at the end of each article to list relevant web links for further consultation by our readers. Could you identify some relevant ones and provide such information as well? Some examples are patient associations, relevant databases, OMIM/proteins/genes links, author's websites, etc...

7) Author contributions: the contribution of every author must be detailed in a separate section.

8) EMBO Molecular Medicine now requires a complete author checklist (https://www.embopress.org/page/journal/17574684/authorguide) to be submitted with all revised manuscripts. Please use the checklist as guideline for the sort of information we need WITHIN the manuscript. The checklist should only be filled with page numbers were the information can be found. This is particularly important for animal reporting, antibody dilutions (missing) and exact values and n that should be indicted instead of a range.

9) Every published paper now includes a 'Synopsis' to further enhance discoverability. Synopses are displayed on the journal webpage and are freely accessible to all readers. They include a short stand first (maximum of 300 characters, including space) as well as 2-5 one sentence bullet points that summarise the paper. Please write the bullet points to summarise the key NEW findings. They should be designed to be complementary to the abstract - i.e. not repeat the same text. We encourage inclusion of key acronyms and quantitative information (maximum of 30 words / bullet point). Please use the passive voice. Please attach these in a separate file or send them by email, we will incorporate them accordingly.

You are also welcome to suggest a striking image or visual abstract to illustrate your article. If you do please provide a jpeg file 550 px-wide x 400-px high.

10) A Conflict of Interest statement should be provided in the main text

11) Please note that we now mandate that all corresponding authors list an ORCID digital identifier. This takes <90 seconds to complete. We encourage all authors to supply an ORCID identifier, which will be linked to their name for unambiguous name identification.

Currently, our records indicate that the ORCID for your account is 0000-0001-9415-346X.

Please click the link below to modify this ORCID:
Link Not Available

12) The system will prompt you to fill in your funding and payment information. This will allow Wiley to send you a quote for the article processing charge (APC) in case of acceptance. This quote takes into account any reduction or fee waivers that you may be eligible for. Authors do not need to pay any fees before their manuscript is accepted and transferred to our publisher.

*Additional important information regarding Figures

Each figure should be given in a separate file and should have the following resolution:
Graphs 800-1,200 DPI
Photos 400-800 DPI
Colour (only CMYK) 300-400 DPI"

Figures are not edited by the production team. All lettering should be the same size and style; figure panels should be indicated by capital letters (A, B, C etc). Gridlines are not allowed except for log plots. Figures should be numbered in the order of their appearance in the text with Arabic numerals. Each Figure must have a separate legend and a caption is needed for each panel.

*Additional important information regarding figures and illustrations can be found at https://bit.ly/EMBOPressFigurePreparationGuideline. See also figure legend preparation guidelines: https://www.embopress.org/page/journal/17574684/authorguide#figureformat

The system will prompt you to fill in your funding and payment information. This will allow Wiley to send you a quote for the article processing charge (APC) in case of acceptance. This quote takes into account any reduction or fee waivers that you may be eligible for. Authors do not need to pay any fees before their manuscript is accepted and transferred to our publisher.

***** Reviewer's comments *****

Referee #1 (Comments on Novelty/Model System for Author):

This is a resubmission. My previous comment on the overall clinical significance has not changed but I recognise that this has been over-ridden by editorial judgement. I respect this decision.

Referee #1 (Remarks for Author):

The authors have addressed my previous comments

Referee #3 (Remarks for Author):

Is suitable for publication

The authors addressed the remaining editorial issues.

5th May 2023

Dear Dr. Hsieh,

We are pleased to inform you that your manuscript is accepted for publication and is now being sent to our publisher to be included in the next available issue of EMBO Molecular Medicine.

Please read below for additional IMPORTANT information regarding your article, its publication and the production process.

Congratulations on your interesting work,

Zeljko Durdevic

Zeljko Durdevic
Editor
EMBO Molecular Medicine

Follow us on Twitter @EmboMolMed
Sign up for eTOCs at embopress.org/alertsfeeds

*** *** *** IMPORTANT INFORMATION *** *** ***

SPEED OF PUBLICATION
The journal aims for rapid publication of papers, using using the advance online publication "Early View" to expedite the process: A properly copy-edited and formatted version will be published as "Early View" after the proofs have been corrected. Please help the Editors and publisher avoid delays by providing e-mail address(es), telephone and fax numbers at which author(s) can be contacted.

Should you be planning a Press Release on your article, please get in contact with embomolmed@wiley.com as early as possible, in order to coordinate publication and release dates.

LICENSE AND PAYMENT:

All articles published in EMBO Molecular Medicine are fully open access: immediately and freely available to read, download and share.

EMBO Molecular Medicine charges an article processing charge (APC) to cover the publication costs. You, as the corresponding author for this manuscript, should have already received a quote with the article processing fee separately. Please let us know in case this quote has not been received.

Once your article is at Wiley for editorial production you will receive an email from Wiley's Author Services system, which will ask you to log in and will present you with the publication license form for completion. Within the same system the publication fee can be paid by credit card, an invoice, pro forma invoice or purchase order can be requested.

Payment of the publication charge and the signed Open Access Agreement form must be received before the article can be published online.

PROOFS

You will receive the proofs by e-mail approximately 2 weeks after all relevant files have been sent o our Production Office. Please return them within 48 hours and if there should be any problems, please contact the production office at embopressproduction@wiley.com.

Please inform us if there is likely to be any difficulty in reaching you at the above address at that time. Failure to meet our

deadlines may result in a delay of publication.

All further communications concerning your paper proofs should quote reference number EMM-2022-16351-V3 and be directed to the production office at embopressproduction@wiley.com.

Thank you,

Zeljko Durdevic
Editor
EMBO Molecular Medicine

EMBO Press Author Checklist

Corresponding Author Name: Sunghoon Kim	
Journal Submitted to: EMBO Molecular Medicine	
Manuscript Number: EMM-2022-16940	

USEFUL LINKS FOR COMPLETING THIS FORM

The EMBO Journal - Author Guidelines
EMBO Reports - Author Guidelines
Molecular Systems Biology - Author Guidelines
EMBO Molecular Medicine - Author Guidelines

Reporting Checklist for Life Science Articles (updated January 2022)

This checklist is adapted from Materials Design Analysis Reporting (MDAR) Checklist for Authors. MDAR establishes a minimum set of requirements in transparent reporting in the life sciences (see Statement of Task: 10.31222/osf.io/9sm4x). Please follow the journal's guidelines in preparing your manuscript.

Please note that a copy of this checklist will be published alongside your article.

Abridged guidelines for figures

1. Data

The data shown in figures should satisfy the following conditions:

- the data were obtained and processed according to the field's best practice and are presented to reflect the results of the experiments in an accurate and unbiased manner.
- ideally, figure panels should include only measurements that are directly comparable to each other and obtained with the same assay.
- plots include clearly labeled error bars for independent experiments and sample sizes. Unless justified, error bars should not be shown for technical replicates.
- if n<5, the individual data points from each experiment should be plotted. Any statistical test employed should be justified.
- Source Data should be included to report the data underlying figures according to the guidelines set out in the authorship guidelines on Data Presentation.

2. Captions

Each figure caption should contain the following information, for each panel where they are relevant:

- a specification of the experimental system investigated (eg cell line, species name).
- the assay(s) and method(s) used to carry out the reported observations and measurements.
- an explicit mention of the biological and chemical entity(ies) that are being measured.
- an explicit mention of the biological and chemical entity(ies) that are altered/varied/perturbed in a controlled manner.
- the exact sample size (n) for each experimental group/condition, given as a number, not a range;
- a description of the sample collection allowing the reader to understand whether the samples represent technical or biological replicates (including how many animals, litters, cultures, etc.).
- a statement of how many times the experiment shown was independently replicated in the laboratory.
- definitions of statistical methods and measures:
  - common tests, such as t-test (please specify whether paired vs. unpaired), simple χ^2 tests, Wilcoxon and Mann-Whitney tests, can be unambiguously identified by name only, but more complex techniques should be described in the methods section;
  - are tests one-sided or two-sided?
  - are there adjustments for multiple comparisons?
  - exact statistical test results, e.g., P values = x but not P values < x;
  - definition of 'center values' as median or average;
  - definition of error bars as s.d. or s.e.m.

Please complete ALL of the questions below.
Select "Not Applicable" only when the requested information is not relevant for your study.

Materials

Newly Created Materials	Information included in the manuscript?	In which section is the information available? (Reagents and Tools Table, Materials and Methods, Figures, Data Availability Section)
New materials and reagents need to be available; do any restrictions apply?	Not Applicable	Data availability

Antibodies	Information included in the manuscript?	In which section is the information available? (Reagents and Tools Table, Materials and Methods, Figures, Data Availability Section)
For **antibodies** provide the following information:<br>- Commercial antibodies: RRID (if possible) or supplier name, catalogue number and or/clone number<br>- Non-commercial: RRID or citation	Yes	Materials and Methods

DNA and RNA sequences	Information included in the manuscript?	In which section is the information available? (Reagents and Tools Table, Materials and Methods, Figures, Data Availability Section)
**Short novel DNA or RNA including primers, probes**: provide the sequences.	Yes	Materials and Methods

Cell materials	Information included in the manuscript?	In which section is the information available? (Reagents and Tools Table, Materials and Methods, Figures, Data Availability Section)
**Cell lines:** Provide species information, strain. Provide accession number in repository **OR** supplier name, catalog number, clone number, and/**OR** RRID.	Yes	Materials and Methods
**Primary cultures:** Provide species, strain, sex of origin, genetic modification status.	Yes	Materials and Methods
Report if the cell lines were recently **authenticated** (e.g., by STR profiling) and tested for mycoplasma contamination.	Yes	Materials and Methods

Experimental animals	Information included in the manuscript?	In which section is the information available? (Reagents and Tools Table, Materials and Methods, Figures, Data Availability Section)
**Laboratory animals or Model organisms:** Provide species, strain, sex, age, genetic modification status. Provide accession number in repository **OR** supplier name, catalog number, clone number, **OR** RRID.	Yes	Materials and Methods
**Animal observed in or captured from the field:** Provide species, sex, and age where possible.	Not Applicable	
Please detail **housing and husbandry conditions**.	Yes	Materials and Methods

Plants and microbes	Information included in the manuscript?	In which section is the information available? (Reagents and Tools Table, Materials and Methods, Figures, Data Availability Section)
**Plants:** provide species and strain, ecotype and cultivar where relevant, unique accession number if available, and source (including location for collected wild specimens).	Not Applicable	
**Microbes:** provide species and strain, unique accession number if available, and source.	Not Applicable	

Human research participants	Information included in the manuscript?	In which section is the information available? (Reagents and Tools Table, Materials and Methods, Figures, Data Availability Section)
If collected and within the bounds of privacy constraints report on age, sex and gender or ethnicity for all study participants.	Not Applicable	

Core facilities	Information included in the manuscript?	In which section is the information available? (Reagents and Tools Table, Materials and Methods, Figures, Data Availability Section)
If your work benefited from core facilities, was their service mentioned in the acknowledgments section?	Not Applicable	

Design

Study protocol	Information included in the manuscript?	In which section is the information available? (Reagents and Tools Table, Materials and Methods, Figures, Data Availability Section)
If study protocol has been **pre-registered, provide DOI in the manuscript**. For clinical trials, provide the trial registration number **OR** cite DOI.	Not Applicable	
Report the **clinical trial registration number** (at ClinicalTrials.gov or equivalent), where applicable.	Not Applicable	

Laboratory protocol	Information included in the manuscript?	In which section is the information available? (Reagents and Tools Table, Materials and Methods, Figures, Data Availability Section)
Provide DOI OR other citation details if **external detailed step-by-step protocols** are available.	Not Applicable	

Experimental study design and statistics	Information included in the manuscript?	In which section is the information available? (Reagents and Tools Table, Materials and Methods, Figures, Data Availability Section)
Include a statement about **sample size** estimate even if no statistical methods were used.	Yes	Materials and Methods
Were any steps taken to minimize the effects of subjective bias when allocating animals/samples to treatment (e.g. **randomization procedure**)? If yes, have they been described?	Yes	Materials and Methods
Include a statement about **blinding** even if no blinding was done.	Yes	Materials and Methods
Describe **inclusion/exclusion criteria** if samples or animals were excluded from the analysis. Were the criteria pre-established?<br><br>If sample or data points were omitted from analysis, report if this was due to attrition or intentional exclusion and provide justification.	Yes	Materials and Methods
For every figure, are **statistical tests** justified as appropriate? Do the data meet the assumptions of the tests (e.g., normal distribution)? Describe any methods used to assess it. Is there an estimate of variation within each group of data? Is the variance similar between the groups that are being statistically compared?	Yes	Figures

Sample definition and in-laboratory replication	Information included in the manuscript?	In which section is the information available? (Reagents and Tools Table, Materials and Methods, Figures, Data Availability Section)
In the figure legends: state number of times the experiment was **replicated** in laboratory.	Yes	
In the figure legends: define whether data describe **technical or biological replicates**.	Yes	

Ethics

Ethics	Information included in the manuscript?	In which section is the information available? (Reagents and Tools Table, Materials and Methods, Figures, Data Availability Section)
Studies involving **human participants**: State details of **authority granting ethics approval** (IRB or equivalent committee(s), provide reference number for approval.	Not Applicable	
Studies involving **human participants**: Include a statement confirming that **informed consent** was obtained from all subjects and that the experiments conformed to the principles set out in the WMA Declaration of Helsinki and the Department of Health and Human Services Belmont Report.	Not Applicable	
Studies involving **human participants:** For publication of **patient photos**, include a statement confirming that consent to publish was obtained.	Not Applicable	
Studies involving experimental **animals**: State details of **authority granting ethics approval** (IRB or equivalent committee(s), provide reference number for approval. Include a statement of compliance with ethical regulations.	Yes	Materials and Methods
Studies involving **specimen and field samples:** State if relevant **permits** obtained, provide details of authority approving study; if none were required, explain why.	Not Applicable	

Dual Use Research of Concern (DURC)	Information included in the manuscript?	In which section is the information available? (Reagents and Tools Table, Materials and Methods, Figures, Data Availability Section)
Could your study fall under dual use research restrictions? Please check biosecurity documents and list of **select agents and toxins** (CDC): https://www.selectagents.gov/sat/list.htm	Not Applicable	
If you used a select agent, is the security level of the lab appropriate and reported in the manuscript?	Not Applicable	
If a study is subject to dual use research of concern regulations, is the name of the **authority granting approval and reference number** for the regulatory approval provided in the manuscript?	Not Applicable	

Reporting

The MDAR framework recommends adoption of discipline-specific guidelines, established and endorsed through community initiatives. Journals have their own policy about requiring specific guidelines and recommendations to complement MDAR.

Adherence to community standards	Information included in the manuscript?	In which section is the information available? (Reagents and Tools Table, Materials and Methods, Figures, Data Availability Section)
State if relevant guidelines or checklists (e.g., **ICMJE, MIBBI, ARRIVE, PRISMA**) have been followed or provided.	Not Applicable	
For **tumor marker prognostic studies**, we recommend that you follow the **REMARK** reporting guidelines (see link list at top right). See author guidelines, under 'Reporting Guidelines'. Please confirm you have followed these guidelines.	Not Applicable	
For **phase II and III randomized controlled trials**, please refer to the **CONSORT** flow diagram (see link list at top right) and submit the CONSORT checklist (see link list at top right) with your submission. See author guidelines, under 'Reporting Guidelines'. Please confirm you have submitted this list.	Not Applicable	

Data Availability

Data availability	Information included in the manuscript?	In which section is the information available? (Reagents and Tools Table, Materials and Methods, Figures, Data Availability Section)
Have **primary datasets** been deposited according to the journal's guidelines (see 'Data Deposition' section) and the respective accession numbers provided in the Data Availability Section?	Yes	Data Availability
Were **human clinical and genomic datasets** deposited in a public access-controlled repository in accordance to ethical obligations to the patients and to the applicable consent agreement?	Not Applicable	
Are **computational models** that are central and integral to a study available without restrictions in a machine-readable form? Were the relevant accession numbers or links provided?	Not Applicable	
If publicly available data were reused, provide the respective **data citations in the reference list**.	Not Applicable	
